# Ultrafast synthesis of carbon quantum dots from fenugreek seeds using microwave plasma enhanced decomposition: application of C-QDs to grow fluorescent protein crystals

**DOI:** 10.1038/s41598-020-69264-9

**Published:** 2020-07-23

**Authors:** Akansha Dager, Ankur Baliyan, Shunji Kurosu, Toru Maekawa, Masaru Tachibana

**Affiliations:** 1 Graduate School of Nanobioscience, Yokohama City University, 22-2 Seto, Kanazawa-ku, Yokohama, 236-0027 Japan; 2 NISSAN ARC, LTD, 1-Natsushima-cho, Yokosuka, 236-0061 Japan; 3 Bio-Nano Electronics Research Centre, Toyo University, 2100, Kujirai, Kawagoe, Saitama 350-8585 Japan

**Keywords:** Quantum dots, Nanoparticles, Quantum dots

## Abstract

Herein, we present the rapid synthesis of mono-dispersed carbon quantum dots (C-QDs) via a single-step microwave plasma-enhanced decomposition (MPED) process. Highly-crystalline C-QDs were synthesized in a matter of 5 min using the fenugreek seeds as a sustainable carbon source. It is the first report, to the best of our knowledge, where C-QDs were synthesized using MPED via natural carbon precursor. Synthesis of C-QDs requires no external temperature other than hydrogen (H_2_) plasma. Plasma containing the high-energy electrons and activated hydrogen ions predominantly provide the required energy directly into the reaction volume, thus maximizing the atom economy. C-QDs shows excellent Photoluminescence (PL) activity along with the dual-mode of excitation-dependent PL emission (blue and redshift). We investigate the reason behind the dual-mode of excitation-dependent PL. To prove the efficacy of the MPED process, C-QDs were also derived from fenugreek seeds using the traditional synthesis process, highlighting their respective size-distribution, crystallinity, quantum yield, and PL. Notably, C-QDs synthesis via MPED was 97.2% faster than the traditional thermal decomposition process. To the best of our knowledge, the present methodology to synthesize C-QDs via natural source employing MPED is three times faster and far more energy-efficient than reported so far. Additionally, the application of C-QDs to produce the florescent lysozyme protein crystals “hybrid bio-nano crystals” is also discussed. Such a guest–host strategy can be exploited to develop diverse and complex "bio-nano systems". The florescent lysozyme protein crystals could provide a platform for the development of novel next-generation polychrome luminescent crystals.

## Introduction

Recently, carbon quantum dots (C-QDs) have gained much attention due to the unique characteristics, notably, alluring fluorescence, chemical-stability, water-solubility, and magnificent photostability properties. C-QDs, owning such properties, have found numerous applications in optoelectronics, bio-imaging, energy-harvesting, and ingenious sensing. Predominantly, C-QDs synthesis is broadly classified into “top-down” and “bottom-up” approaches^1,2^. In the top-down approach, C-QDs are synthesized via employing the arc discharge, laser ablation, and chemical oxidation techniques that essentially disintegrate the large graphitic carbon materials into smaller ones^3–5^. Alternatively, in the bottom-up approach, C-QDs are synthesized what is known as chemical synthesis such as; the thermal decomposition^6^, hydrothermal^7^, electrochemical oxidation^8^, and microwave pyrolysis^9–12^. The majority of the synthesis processes are usually energy consuming, pretty cumbersome, and demand expensive carbon sources that are often toxic^12–16^. Concerning C-QDs synthesis, conventional methods such as thermal decomposition, solvothermal and hydrothermal require a higher temperature, longer operational time, and complicated experimental set-up^17^. Alternatively, microwave pyrolysis has a distinct disadvantage that the incident energy is not directly transferred to the carbon precursor. Thus C-QDs often have an amorphous structure and poor photostability. Recently, our group reported the synthesis of excitation independent C-QDs; however, the synthesis process was time and energy-consuming^18^. Despite recent advancements in the synthesis technologies, developing a cost-effective, nonetheless rapid, and energy-efficient process remains a challenge to synthesize graphitized-C-QDs.

Considering the nanomaterial’s synthesis, chemical vapor deposition (CVD) and plasma-enhanced chemical vapor deposition (PECVD) were widely used to synthesize verities of carbon nanomaterials; carbon nanotubes (CNTs), carbon nanowalls (CNWs) and few-layer graphene (FLG)^19–22^. The available literature on the synthesis of C-QDs by CVD is highly scarce, and only a single report is available^23^. Although, plasma has numerous advantages compared to synthesis methods such as pyrolysis, hydrothermal, and microwave pyrolysis^6–8^. It is worth mentioning that there is no report on the synthesis of C-QDs employing microwave plasma-enhanced decomposition (MPED). Indeed, CVD is not the obvious choice for the synthesis of C-QDs because it requires high temperature, prolonged reaction-time, and expensive precursors^23^. Conversely, MPED has no such limitations thanks to the plasma-enhanced decomposition that harnesses the power of plasma. In MPED, high-energy electron/charged ions require far less energy for the synthesis of C-QDs. The growth of carbon nanomaterials is very rapid^19^. Additionally, MPED is similar to the PECVD process except that the precursors can be any natural carbon source and can be used in the as-is form. Inspired by the PECVD versatility, it would be interesting to synthesize C-QDs using MPED that required almost no change in conventional PECVD set-up and the prerequisite of the carbon feedstock gases can be excluded^19^.

C-QDs have plenty of applications but rarely used to grow the florescent protein crystals. As per our knowledge, C-QDs have never been incorporated into the lysozyme protein crystal. The guest–host strategy, i.e., C-QD incorporation into the lysozyme protein crystals, can be exploited to make the luminescent bio-nano hybrid systems. Lysozymes protein is a porous material and possesses solvent channels. Lysozyme’ pores filled with aqueous buffer solutions provide templates to grow nanomaterials and encapsulate the desired guest molecules^24,25^. Encapsulation of the desired guest molecules can be done either via a solution or crystal directed approach. The solution directed approach is widely tested, which includes protein conjugation with inorganic-QDs. Such bio-nano hybrids maintain their biological activities and find applications in bio-targeting^26,27^. Encapsulation of the desired guest molecules in the proteins was mostly explored by the solution directed approach. Even though the solution directed approach is impressive; however, the crystal directed approach is more versatile and promising to study the complex mechanisms compared to the solution directed approach^28,29^. Inspired by this fact, hosting the C-QDs into the lysozyme protein channels will be fascinating.

Herein, for the first time, we report the rapid synthesis of mono-dispersed C-QDs using MPED. Considering the sustainable carbon source, fenugreek seeds (*Trigonella foenum-graecum*) were used as a carbon source for the synthesis of C-QDs. Fenugreek seeds are eco-friendly (antibacterial & antioxidant), high abundance, and economical. Moreover, unlike the fruits juices and peels, fenugreek seeds do not undergo seasonal fluctuation and can be stored over a period of time. Fenugreek seeds are mainly composed of carbohydrates and proteins that provide necessary carbon source for the synthesis of C-QDs. MPED is a rapid and energy-efficient process that requires no external temperature, and high-quality C-QDs can be synthesized in a matter of 5 min. Hydrogen (H_2_) plasma, containing the high-energy electrons/hydrogen ions, predominantly provide the required energy, upon striking on to the fenugreek powder, thus maximizing the atom economy; minimizes the energy requirement, and shortens the reaction time. As a result, the necessity of external temperature was avoided. To prove the efficacy of purposed MPED, C-QDs were also synthesized using fenugreek seeds employing conventional thermal decomposition process, and their respective size-distribution, crystallinity, and photoluminescence (PL) properties are discussed. Additionally, the guest–host strategy was used to produce the C-QDs based florescent lysozyme protein crystals "hybrid bio-nano crystals". Such a strategy can be exploited to develop next-generation polychrome luminescent crystals.

## Experimental methods

### Materials

Fenugreek seeds were procured from (Swati seeds, India) and were used in the as-is form, i.e., without any further purification. Throughout the experiments, scientific grade deionized water (DI) was used.

### Synthesis of C-QDs

**Figure 1:**
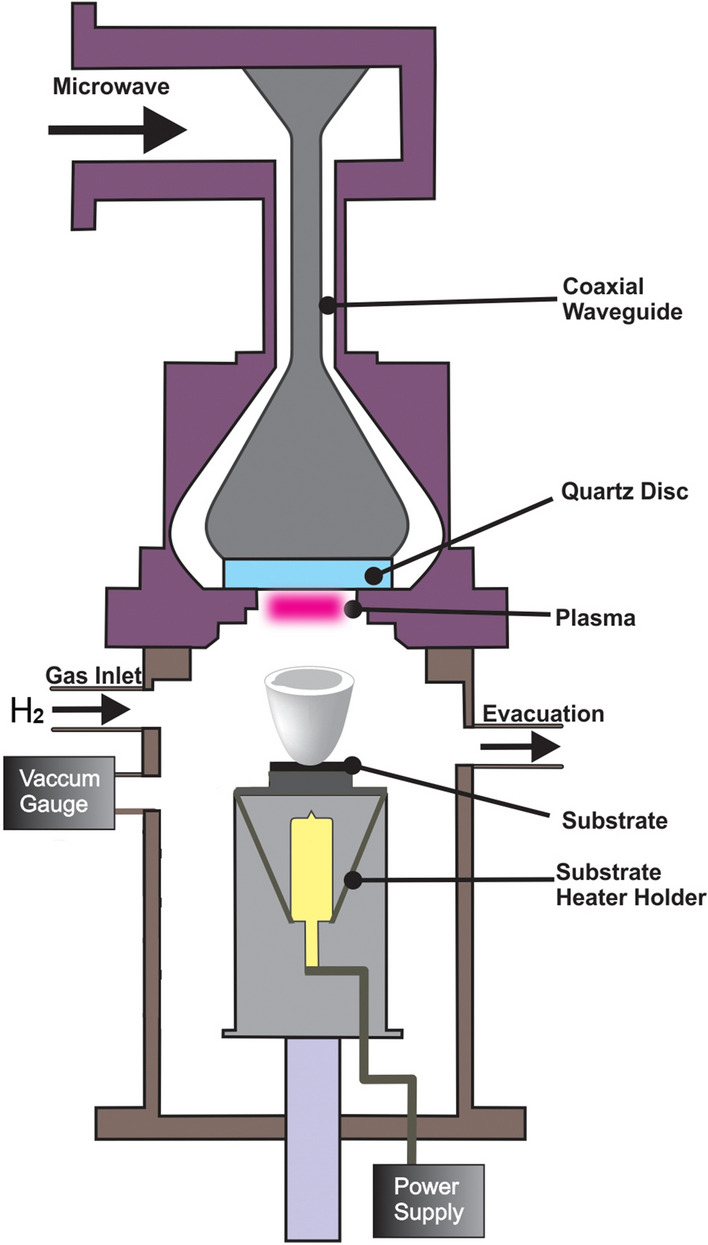
The schematic diagram of the MPED set-up.

At first, as received, fenugreek seeds were crushed with a mixer grinder (Tiger mixer grinder, Japan). Subsequently, ground greenish-fenugreek seed-powder (0.2 g) was transferred to the crucible cup (AS ONE, Japan). Figure 1 shows the schematic illustration of the MPED^19^ set-up (ULVAC, Inc). Crucible cup, containing the ground fenugreek powder, was mounted on to the MPED substrate holder. For the ignition of plasma, the microwave power was set at 500 W. The gap between the plasma and the crucible cup was fixed at 40 mm. Hydrogen (H_2_) was introduced into the MPED chamber at a constant flow rate of 30 standard cubic centimeters per minute (sccm) for 5 min. During the entire synthesis process, the pressure of chamber was maintained at 30 Pa (0.23 Torr). Predominately, no external-temperature was given to the substrate holder. However, the thermocouple attached to the substrate holder shows that the temperature was below 70 °C.

After the MPED, the greenish fenugreek powder turned dark gray. The carbonized gray-product was dispersed in DI and sonicated for 5 min. The black color suspension was centrifuged at 15,000 rpm for 10 min to remove the large un-dissolved particles. Thereafter, the supernatant containing the C-QDs was filtered using pore size filter (100 nm, PALL ACRO DISC, Japan). For further purification, the dialysis was performed via a dialysis kit (Float-A-Lyzer G2 Dialysis, Japan)^18^. Lastly, the purified C-QDs were transferred to the glass vial and stored for further characterization. The synthesis of C-QDs using thermal decomposition is explained in the supplementary information (SI). Hereafter, for ease, the C-QDs synthesized with MPED and thermal decomposition methods are referred with C_PE_-QDs and C_PY_-QDs, respectively.

### Characterization of C-QDs

The optical properties of C-QDs were investigated by using photoluminescence spectroscopy (FP-6500, JASCO, Japan) and UV–Vis absorption spectroscopy (V-530, JASCO, Japan). The size-distribution and surface-charge of C-QDs were estimated by Zeta-sizer (Nano-ZS 90 Zetasizer, Malvern Instruments Ltd). The diameter distribution and structure of C-QDs were also ascertained by transmission electron microscopy (TEM, JEM-2200FS, JEOL). The purity of the C-QDs was ascertained by Thin-layer chromatography (TLC) analysis using the TLC Silica gel 60 F254 plate (Merck Millipore). The structural purity and elemental composition of C-QDs were characterized by laser Raman spectroscopy (Lab RAM, HR-800, Horiba JOBIN YVON S.A.S.) and energy-dispersive X-ray spectroscopy (EDS, JEM-2300F, JEOL), respectively. To determine the functional groups on to the surface of C-QDs, the Fourier transform infrared spectra (FTIR) of dried C-QDs were recorded (FTIR-4100, JASCO, Japan). In order to determine the chemical state of as-synthesized C-QDs, the X-ray photoelectron spectroscopy analysis was done (PHI Quantes, ULVAC-PHI. Inc.). The quantum efficiency measurement system (QE-2000, Otsuka Electronics, Japan) was used to investigate the quantum yield of C-QDs.

### Synthesis of florescent lysozyme protein crystals

Hanging drop method was used to grow the florescent lysozyme protein crystals. At first, Lysozyme (400 μg) and C_PE_-QDs (20 μg dispersed in 5 ml distilled water) resulting in 84 mg/ml C-QD-lysozyme solution were prepared. Simultaneously, a precipitant solution (sodium acetate buffer 1.0 M NaCl in 100 mM, pH—4.5) was also prepared. The C-QD-lysozyme solution and precipitant solution were mixed in equal volumes. The resulting crystallization-solution was filtered (pore size of 0.2 μm) to remove large protein aggregates or foreign particles. A 24 well-tray filled with reservoir precipitant solution 2 ml/well, was utilized to hold the reservoir precipitant. The inverted coverslip containing the protein crystallization solution (20 μl/coverslip) was placed on to the reservoir well and sealed by the vaseline grease to create a closed system. The well-tray was placed at the incubation temperature of 21 °C for three days. In parallel, following the same protocol as described above, the control lysozyme protein crystals (without C-QDs) were also prepared. Hereafter, for simplicity, the pure lysozyme protein crystals (control), and C-QDs doped-lysozyme protein crystals will be addressed as Lysozyme and CQD-Lysozyme protein crystals, respectively.

### Characterization of protein crystals

The surface morphology, shape, and size of lysozyme and CQD-Lysozyme protein crystals were observed with a digital optical microscope (Nikon SMZ1500). The fluorescent behavior of Lysozyme and CQD-Lysozyme crystals were investigated with a fluorescent confocal microscope (Leica DMI6000).

## Results and discussion

PECVD has been extensively used to grow common carbon nanostructures at a far lower temperature than chemical vapor deposition^30^. Our group has used PECVD to synthesize the vertically aligned carbon nanotubes and carbon nanowalls^19–21^. Due to the higher electron and ion density, the PECVD becomes more attractive when operated at low-pressure (0.1–0.5 Torr) operation^31^. It is essential to explore the discharge diagnostics of plasma being used for the synthesis of nanostructures. The optical emission spectrum (OES) of the excited hydrogen plasma is shown in Figure S1†. The OES results show that peaks at 658.2, 486.9, 434.7, and 463.8 nm can be ambiguously assigned to H_α_, H_β,_ H_γ_, and secondary hydrogen, respectively. Fulcher band has much lower intensities that indicate that the plasma has a high degree of H_2_ dissociation^32,33^. As illustrated in Figure S1†, the OES spectra show that the abundant charge ions in the hydrogen plasma can be utilized to reduce the natural carbon source to synthesize the C_PE_-QDs.

Primarily, the formation of C_PE_-QDs consists of steps of carbon precursor breaking, nucleation, and growth. At first, the hydrogen gas (H_2_) introduced into the MPED chamber, and H_2_ gets ionized with plasma exposure; it is worth noting that no external temperature was provided to the substrate holder. Fenugreek seeds are mainly composed of carbohydrates and proteins. Carbohydrates are vital ingredient that provides necessary carbon for the synthesis of C-QDs^34^. By virtue of denser plasma, hydrogen ions, and abundant high-energy electrons with high kinetic energy strike on the ground fenugreek powder and thereby exchange momentum before coming to rest. While doing so, enough energy is transferred to the fenugreek molecular precursor. The carbon source breaks down into the atomic species; carbon, hydrogen, and oxygen atoms. In the nucleation stage that follows, the carbon species come together possibly by diffusion and form smaller nuclei. These smaller carbon nuclei continue to grow as the time progresses with the availability of new carbon atoms forming the C_PE_-QDs via diffusion. Therefore, the growth of C_PE_-QDs continues until there is not enough carbon nearby that can continue to feed the new carbon atoms to the C_PE_-QDs, and the growth of C_PE_-QDs comes to a halt.

**Figure 2:**
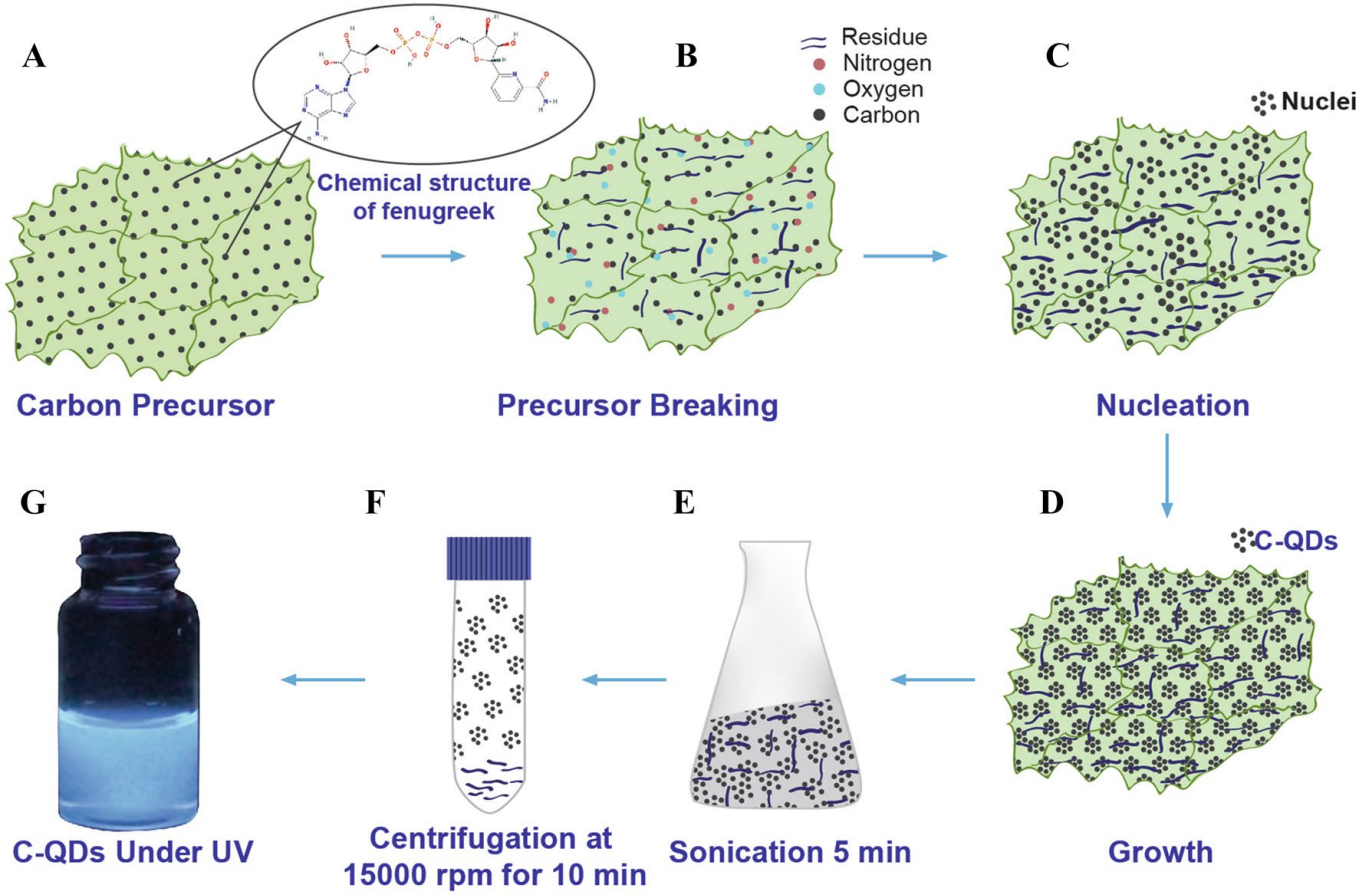
The schematic illustration shows the synthesis of C-QDs from fenugreek seed via MPED method. (**A**) Carbon precursor (crushed fenugreek seeds), (**B**) Carbon precursor breaking via MPED, (**C**) Nucleation of the C-QDs at early stage of MPED, (**D**) Growth of the C-QDs after 5 min of MPED, (**E**) Sonication of C-QDs, (**F**) Centrifugation of C-QDs, (**G**) C-QDs under UV.

**Figure 3:**
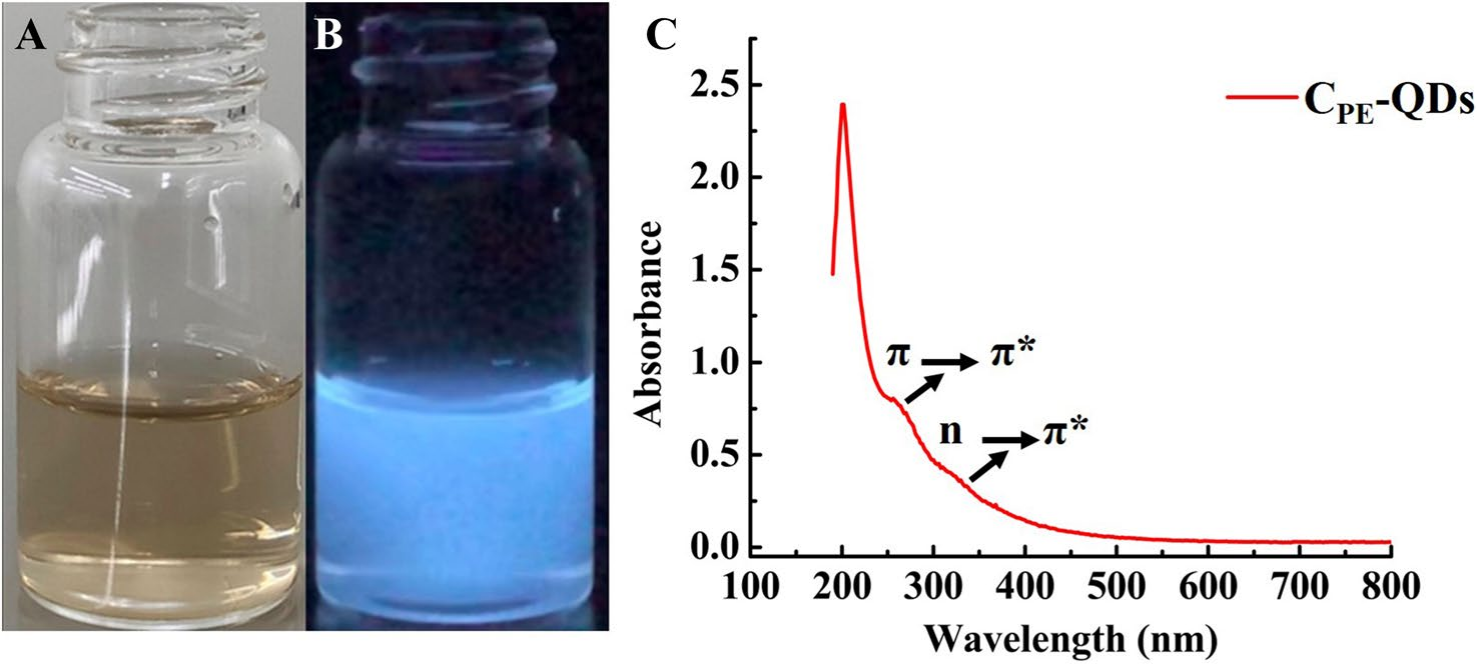
(**A**) Optical images of C_PE_-QDs dispersed in water under the regular daylight exposure. (**B**) Optical images of C_PE_-QDs dispersed in water under UV exposure (365 nm). (**C**) UV–Vis absorption spectra of as-synthesized C_PE_-QDs.

The schematic diagram of carbon quantum dots synthesis via the MPED process is illustrated in Fig. 2. The schematic illustration shows six steps involved for the synthesis of C-QDs; (1) carbon precursor (crushed fenugreek seeds), (2) breaking the carbon precursor via MPED, (3) nucleation of the C-QDs at the early stage of MPED, (4) growth of the C-QDs, (5) sonication of C-QDs, (6) centrifugation of C-QDs. The optical image of C_PE_-QDs dispersed in water under normal light, and UV light is shown in Fig. 3A,B, respectively. The C_PE_-QDs suspended in water shows strong bluish fluorescence emission under UV exposure (excitation 365 nm). The absorption spectra of the as-prepared C_PE_-QDs is shown in Fig. 3C, exhibit peaks at 270 and 338 nm, ambiguously ascribed to the π–π* transition of C=C and n–π* transition of C=O bonds, respectively^8,35^.

**Figure 4:**
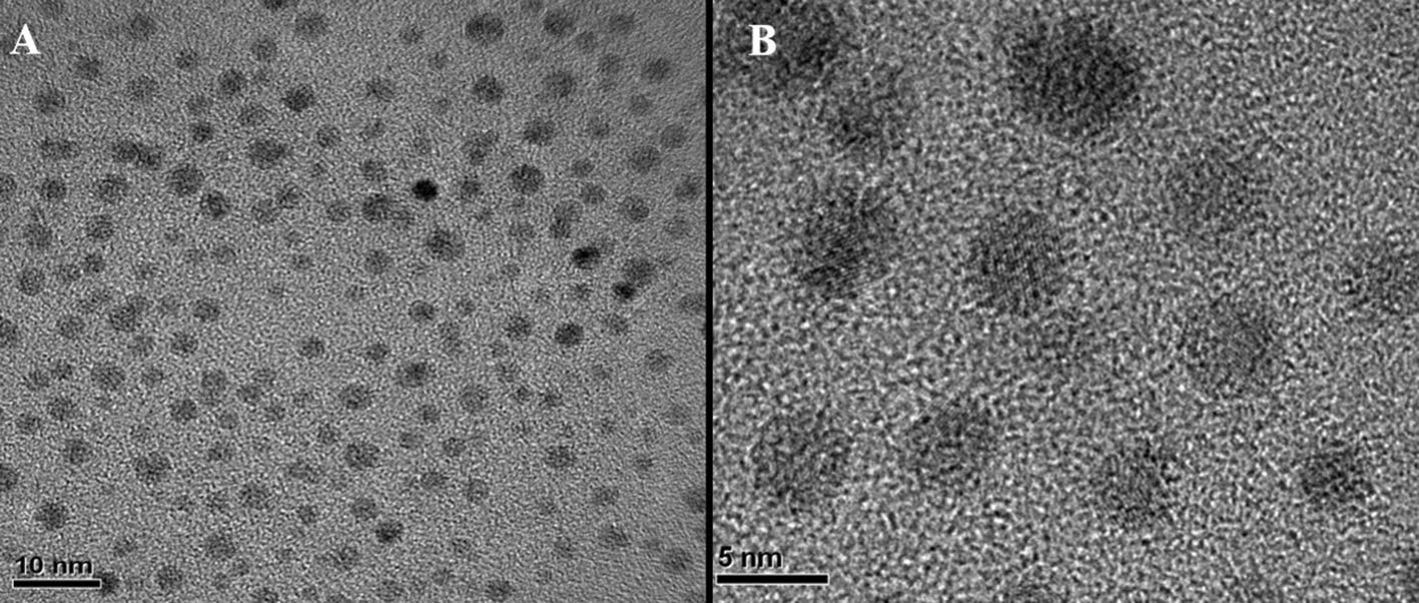
TEM images of as-synthesized C_PE_-QDs. (**A**) C_PE_-QDs at low resolution. (**B**) C_PE_-QDs at high resolution, crystalline lattices can be seen.

The low and high-resolution TEM images of C_PE_-QDs are shown in Fig. 4A,B, respectively. Sixty-five high-resolution TEM images of C_PE_-QDs were recorded by scanning the random positions on to the TEM grid for the precise diameter distribution of C-QDs. Cumulatively, one hundred thirty-four (134) C_PE_-QDs nanoparticles were taken into account to evaluate the average-diameter distribution of C-QDs. The as-synthesized C_PE_-QDs were uniform in size (Fig. 4A). C_PE_-QDs were highly mono-dispersed with an average size diameter of 4.25 ± 0.56 nm (Figure S2† for the distribution of the diameters of C_PE_-QDs). The lattice spacing in the C_PE_-QDs was found to be 0.21 nm. It was assigned to (100) plane of carbon nanoparticle (Figure S3† for high-resolution lattice spacing).^27^ Clearly visible lattice fringes within the particles show that C_PE_-QDs were crystalline. Often, C-QDs synthesized from natural carbon sources resulted in the amorphous carbon structure^15,26^. However, the crystalline structure of the C_PE_-QDs in the present report is because of the sufficient graphitization as confirmed by TEM images. A point beam EDS spectrum shows that the C_PE_-QDs consist of only carbon and oxygen peaks. EDS results indicate no elemental signature of any foreign impurities other than carbon and oxygen on the TEM grid. It means that the synthesized C_PE_-QDs have no foreign impurities (Figure S4† for the point-beam EDS spectrum of C_PE_-QDs). Although, it is indispensable to distinguish whether the carbon peak was originated from the micro-grid supported carbon-film (TEM grid) or the C_PE_-QDs sample itself, or is it the superimposition of signals from the C_PE_-QDs and TEM grid. Nevertheless, the objective of the EDS was just to ensure that the C_PE_-QDs sample does not contain any residual foreign-element other than carbon and oxygen on the grid^13^.

**Figure 5:**
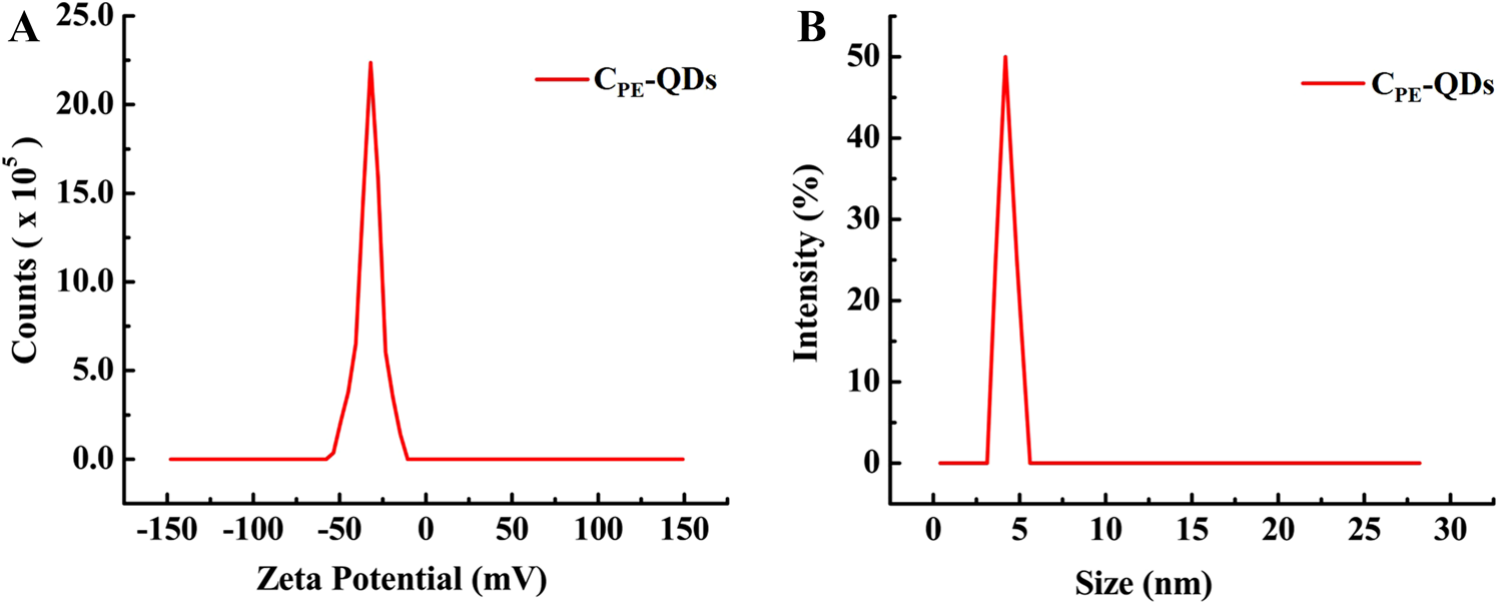
(**A**) Dynamic light scattering (DLS) size distribution curve of C_PE_-QDs dispersed in water. (**B**) The zeta-potential curve of C_PE_-QDs dispersed in water, and the curve shows that C_PE_-QDs have a negative surface charge.

The size distribution of as synthesize C_PE_-QDs was verified by zeta-seizer, as shown in Fig. 5A. The average size of the C_PE_-QDs was found to be 4.37 ± 0.41 nm, which almost correlated with the results obtained with TEM; that is 4.25 nm. It is noteworthy that the hydrodynamic diameter of a particle in the solvent is, in general, larger than the size of C_PE_-QDs measured in vacuum. C-QDs without having any functional group on its surface can precipitate easily if dissolved in aqueous-based solvent given the fact that the bare C-QDs are hydrophobic. The multiple functional groups over the C-QDs surface improve their dispersion in a water-based media; that is why the functionalization of C-QDs is significantly essential. Therefore, the zeta-potential measurement was done to ascertain the polarity of the surface charge that exists on the surface of C_PE_-QDs. Figure 5B, Zeta-potential of the C_PE_-QDs, depicts a narrow-peak at negative 32 mV having a peak-width of 14.8 mV. The negative potential is referred to as the existence of negatively-charged moieties attached to the surface of C_PE_-QDs. Such negatively charged moieties are essential for excellent dispersion of C_PE_-QDs in aqueous-based solvent. As a matter of fact, storing the C_PE_-QDs at room temperature for ten months shows no sign of turbidity, it indicates that the colloidal stability of C_PE_-QDs is sufficiently high and can be used over a period of time as and when needed (Figure S5† fresh C_PE_-QDs and C_PE_-QDs stored for six months).

**Figure 6:**
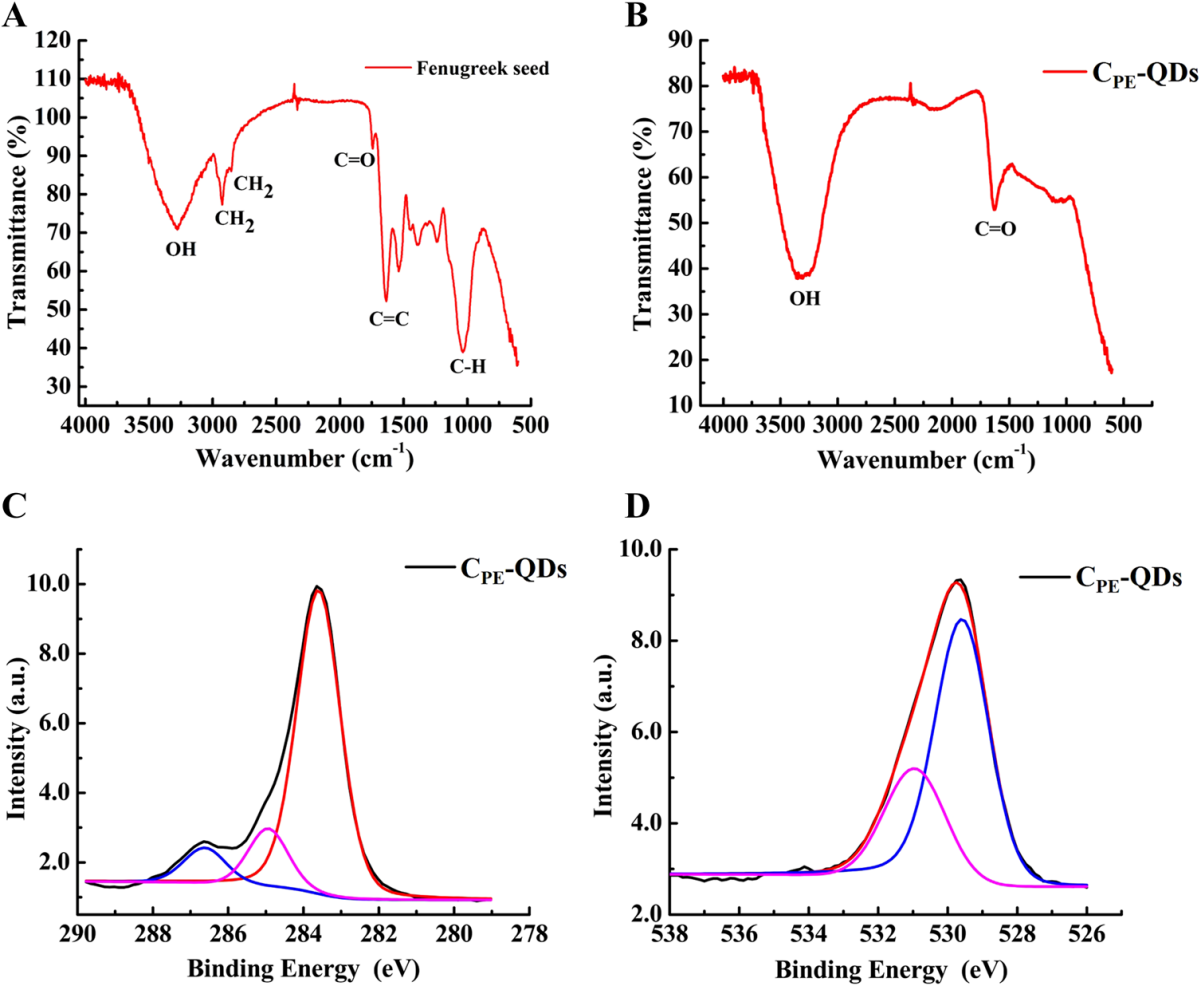
(**A**) FTIR spectra of ground fenugreek seeds (carbon precursor for the synthesis of C_PE_-QDs), (**B**) FTIR spectra of as-synthesized C_PE_-QDs, Carbon quantum dots have mainly the C=O and C=O peaks, (**C**) XPS spectra of C 1*s* spectrum of as-synthesized C_PE_-QDs, the spectrum was de-convoluted into three significant peaks at 283.60 (C-*sp*^2^), 285.09 (C–O) and 286.79 eV (C=O), respectively, (**D**) XPS spectra of O 1*s* spectrum of as-synthesized C_PE_-QDs, the spectrum was de-convoluted into two significant peaks at 529.58 (C=O) and 531.01 eV (C–O) respectively.

It is worth noting that the zeta potential of C_PE_-QDs affirms the presence of negative charge moieties on the surface of C_PE_-QDs. Nevertheless, the chemical structure of moieties cannot be determined by lone zeta potential characterization. Therefore, FT-IR analysis of prepared C_PE_-QDs assists in determining the possible chemical structure of the moieties. The FT-IR spectrum of fenugreek seeds and as prepared C_PE_-QDs are shown in Fig. 6A,B, respectively. The FT-IR spectra of fenugreek seeds have significant peaks at 3,283, 2,925, 2,855, 1,740, 1638, and 1,032 cm^−1^ were assigned to vibrations OH stretching, symmetric CH_2_, asymmetric CH_2_, carboxyl/carbonyl C=O, C=C and C–H bending, respectively^13^. The symmetric and asymmetric CH_2_ peaks in fenugreek FT-IR spectra at 2,925 and 2,855 cm^−1^, respectively, signifies that the fenugreek seeds were predominantly composed of carbohydrate^36^. On the contrary, FT-IR spectra of C_PE_-QDs have significant peaks at 3,323, and 1,630 cm^−1^ can be assigned to OH and C=O functional groups, respectively^37^. The presence of C=O and OH peaks evinces that the C_PE_-QDs have carbonyl and hydroxyl surface moieties. Zeta-potential and FT-IR results complement each other, established the fact that the surface of as-synthesized C_PE_-QDs has intrinsically negatively-charged functional groups attached to the surface of C_PE_-QDs that are beneficial for long term colloidal stability.

Occasionally, a certain proportion of the functional group, due to insufficient carbonization, from the primary “carbon sources” still be present on the surface/interior of as-synthesized C-QDs^38^. For instance, such insufficient carbonization is the solo reason to yield poor photoluminescence^38^. However, by MPED, 5 min of MPED reaction time was sufficient for the carbohydrates (Fenugreek powder) to be carbonized and transform them into the well graphitic C-QDs. Herein, the absence of asymmetric and symmetric peaks of CH_2_ in C_PE_-QDs FT-IR spectra at 2,855 and 2,925 cm^−1^, respectively, predominantly indicate that the carbon source (fenugreek seeds) get fully carbonized after 5 min of MPED. In contrast, in the case of thermal decomposition process, the complete carbonization of ground Fenugreek powder could be achieved only after continuous pyrolysis of the Fenugreek powder at 500 °C for 3 h and holding it more extended period has not resulted into any substantial change in either PL or crystallinity (results are not shown).

XPS analysis of as-synthesized C_PE_-QDs and ground fenugreek seed is shown in Figure S6A† and S6B†, respectively. The survey scan of C_PE_-QDs XPS shows that the carbon quantum dots are composed of mainly carbon, nitrogen and oxygen; i.e., C1*s* (63.42 at.%), N1*s* (5.6 at.%) and O1*s* (31.0 at.%), respectively (Table S1†). Since no trace of any other residual element was detected, that illustrates the purity of the as-synthesized C_PE_-QDs. One out of every three-carbon atoms were functionalized with oxygen that depicts the C_PE_-QDs have a high degree of functionalization. Deconvolution of C1*s* peak is shown in Fig. 6C, the main peak at 283.6 eV (77.5 at.%), which corresponds to the *sp*^2^ graphitic structure. In contrast, the peak at 285.0 (13.5 at.%) and 286.8 eV (8.9 at.%) is attributed to C–O and C=O, respectively (Table S2†). Indeed, XPS results complement the FT-IR results, there too primarily C=O, and C–O bonds were detected. Deconvolution of oxygen peak is shown in Fig. 6D, peak at 529.5 eV (65.9 at.%) is attributed to OH/C=O on the surface of C_PE_-QDs, whereas the peak at 531.0 eV (34.1 at.%) are originated from the functional group; C–O (Table S3†)^39^. The deconvolution of C_PE_-QDs XPS spectra of N 1*s* in Figure S7† reveals two significant peaks at 399.03 (14.1 at.%) and 401.21 eV (85.9 at.%) corresponds to the Pyridinic-N and Graphitic-N, respectively (Table S4†).

XPS helps to understand the underlying transformation from biomaterials “fenugreek seeds” to the C_PE_-QDs. Quantitative XPS analysis of fenugreek seeds for survey scan, C 1*s*, O 1*s*, and N 1*s* are summarized in Table S5†, S6†, S7†, and S8†, respectively. The deconvolution of C 1*s*, O 1*s*, and N 1*s* shown in Figure S8A†, S8B†, and S8C†, respectively, verify the fact that fenugreek seeds were mainly composed of carbohydrates and proteins^34^. Two out of every three-carbon atoms of fenugreek seeds were functionalized with oxygen. However, after the MPED, the amount of oxygen was reduced, whereas the carbon and nitrogen contents were increased. It validates the assumption that the carbonization of fenugreek seeds takes place because of plasma, and as a result, the biomaterials transformed into C_PE_-QDs. After the MPED process, the C_PE_-QDs were found to be intrinsically doped with N-atoms, XPS analysis revealed that N-atoms state of Fenugreek seeds (Table S8† and Figure S8C†) eventually turned into Pyridinic-N and Graphitic-N (Figure S7†).

**Figure 7:**
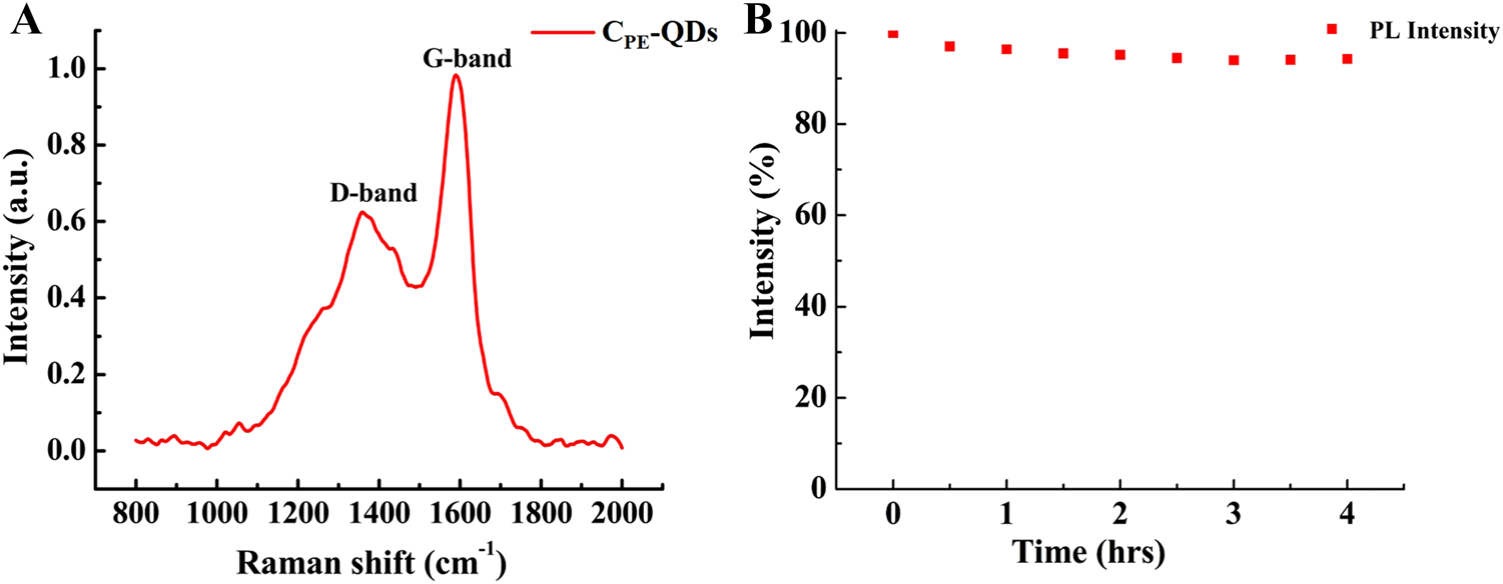
(**A**) Raman spectra of as-synthesized C_PE_-QDs (excitation wavelength 785 nm) shows G-band (1,590 cm^−1^) and D-band (1,362 cm^−1^). (**B**) Photoirradiation stability test of C_PE_-QDs shows that continuous irradiation from a xenon lamp of 150 W for 4 h attribute almost no meaningful reduction in the PL intensity.

Raman spectrum of as-synthesized C_PE_-QDs is shown in Fig. 7A (excitation wavelength 785 nm). Raman spectrum shows two distinct peaks at 1,362 (D-peak) and 1,590 cm^−1^ (G-peak). The G-peak in Raman spectrum (*sp*^2^ hybridization) corresponds to the amount of graphitization associated with the C_PE_-QDs, and D-peak (*sp*^3^ hybridization) illustrates the contribution from the amount of defects, edge effect, and functionalization. The intensity ratio of the G peak to the D peak for C_PE_-QDs specimen was turned out to be 1.68, and this ratio is slightly higher than the C-QDs synthesized by other natural carbon sources.^15,35,36^ The higher G/D ratio emphasizes that the as-synthesized C_PE_-QDs are composed of crystalline graphitic structure. HRTEM results (Figure S3†) also complement the Raman results. The XPS analysis, Raman spectrum and IR spectrum suggest that C_PE_-QDs have close resemblance with oxidized graphite or graphene oxide (GO)^40,41^. However, C_PE_-QDs cannot be either oxidized graphite or GO because of the following three reasons; (1) C_PE_-QDs have spherical architecture (TEM), (2) In case of GO, the ration of (C=O) to *sp*^2^ graphitic structure is very large (XPS of C 1*s*), however, for C_PE_-QDs this ration is not so large, and (3) Oxidized graphite/GO have very narrow PL spectra, unlike to the C_PE_-QDs’ broad PL peak observed in the present report^42–44^.

As shown in Fig. 7B, the photoluminescence of C_PE_-QDs is remarkably stable under photoirradiation exposure. The continuous irradiation attributed no meaningful decline in the PL intensity (Xenon lamp of 150 W for 4 h), and that illustrates the excellent photostability of as-synthesized C_PE_-QDs^10,42,43^. It is believed that unlike the amorphous C-QDs, better crystalline structure and thorough carbonization of C_PE_-QDs might be the rational explanation for excellent photostability of as synthesis C_PE_-QDs^44^.

The quantum yield (QY) of as-synthesized C_PE_-QDs was (excitation wavelength 340 nm) was estimated to be 4.96%; however, the QY of C_PE_-QDs was almost half in comparison to the fennel derived C-QD_S_. Single molecular C-QD tracking studies and adequate purification is necessary to accurately explain what might be the origin of the PL mechanism in CDs^45^. PL mechanisms are constantly under debate. Gan et al. have provided an excellent review of the possible excitation-dependent and excitation-independent PL mechanism^46^. Surface engineering (surface-state), altering the degree of carbonization and shape of C-QDs (hollow interiors), was the rational explanation for excitation-independent PL^46^. In contrast, quantum confinement, charge transfer, surface traps, intramolecular H-bonds, aromatic molecules, the electronegativity of heteroatoms, and synergistic models were purposed to explain the excitation-dependent PL^9,35,36,46^. Nevertheless, transitions from the multiple surface states and bandgap transition caused by the quantum confinement^35^ are widely accepted excitation-dependent PL mechanisms^46,47^.

**Figure 8:**
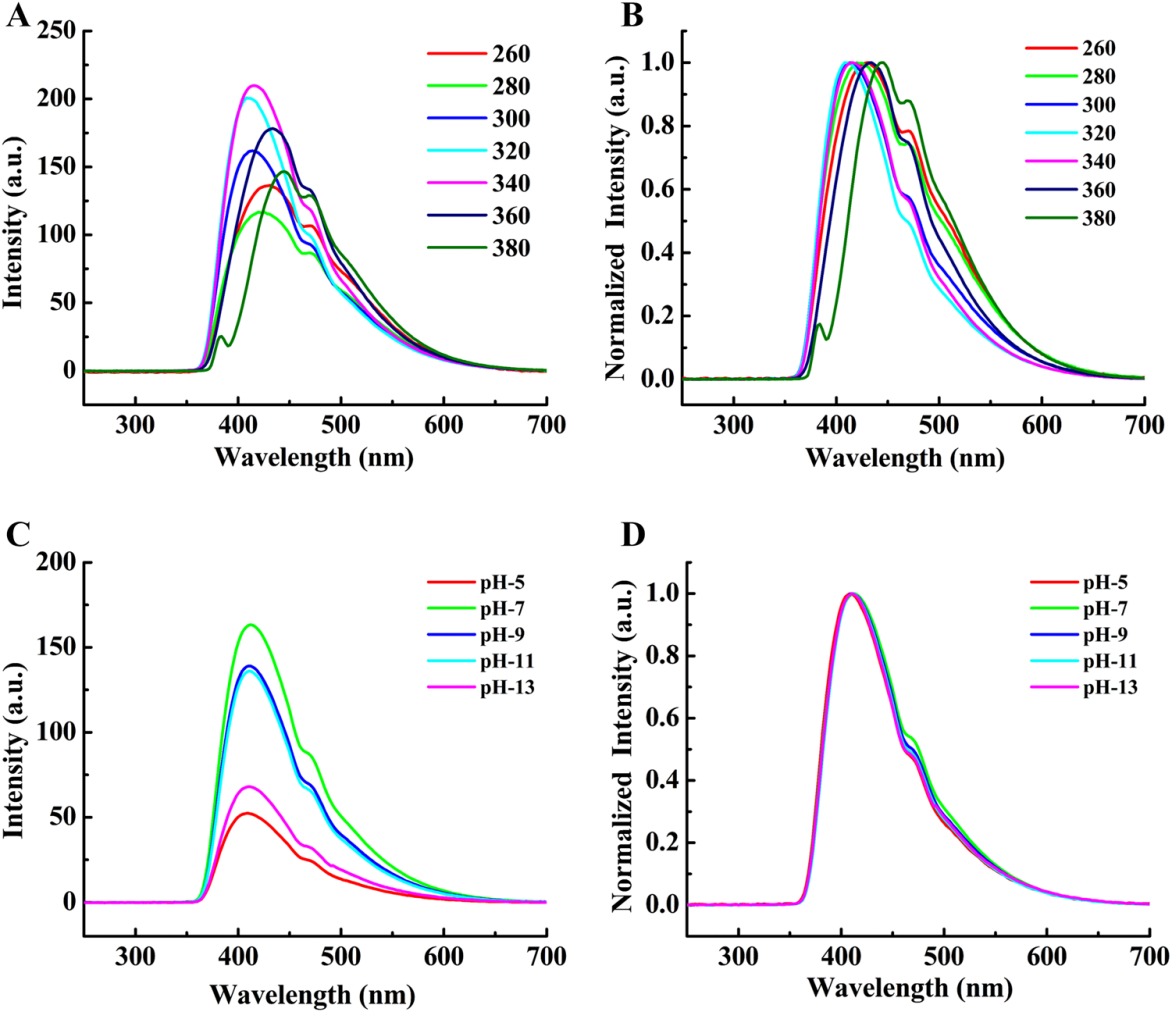
(**A**) PL emission spectra of C_PE_-QDs excited at various energies (260, 280, 300, 320, 340, 360 and 380 nm, C_PE_-QDs were found to be dependent on the excitation energy, (**B**) Normalized PL emission spectra of C_PE_-QDs excited at various energies (260, 280, 300, 320, 340, 360 and 380 nm, shows the dual mode (blue and redshift), (**C**) PL emission spectra of C_PE_-QDs dispersed in water at various pH (acidic to basic) (excitation wavelength 320 nm), (**D**) Normalized PL emission spectra of C_PE_-QDs dispersed in water at various pH (acidic to basic) (excitation wavelength 320 nm), C_PE_-QDs were found to be independent of the excitation energy.

Figure 8A shows the PL of C_PE_-QDs dispersed in water at various excitation; 260, 280, 300, 320, 340, 360, and 380 nm, respectively. The PL emission spectra of C_PE_-QDs was found to be excitation-dependent and had a broad asymmetric peak ranged from 357 to 665 nm. In fact, the maximum emission intensity of C_PE_-QDs was recorded at excitation 340 nm. The peak fitting shows that a typical PL emission spectrum is composed of two peaks; (1) the main peak centered at 414 nm (FWHM 46 nm), and (2) another shoulder peak at 468 nm (FWHM 88 nm) (Figure S9†). It is noteworthy that the excitation-dependent C-QDs can be readily synthesized from the synthetic and natural carbon precursors, and, for instance, C-QDs always shows the redshift.^2,11,42^ Similarly, C_PE_-QDs synthesized from the natural carbon source in the present report also shows that the C_PE_-QDs is excitation-dependent^6,9,10,14,35,36,46,47^^.^

Understanding the origin of PL in C-QDs is a strategic asset for various applications. Surprisingly, as synthesized C_PE_-QDs shows the dual-mode (blue and redshift) of excitation-dependent photoluminescence^48^ (Figure S10†). For instance, the blue-shift (22 nm) as the excitation wavelength changes from 260–320 nm and redshift (40 nm) when C_PE_-QDs were excited with 340–380 nm; the total shift (blue + red) was found to be 62 nm (Fig. 8B for normalized spectra of C_PE_-QDs excitation at different wavelengths). Although the redshift with an increasing excitation wavelength can be explained with quantum confinement,^46^ nevertheless, the existence of blue-shift indicates that the quantum confinement cannot be the lone possible factor for the PL^6,9,10,14,35,36,49,50^. Furthermore, C_PE_-QDs have C–O, and C=O functional groups with moderate functionalization (< 24%), and, therefore, energy transitions from the multiple surface state possibly might also be contributing to the photoluminescence.

Essentially, we believe that two or more fluorescence mechanisms probably active and might be contributing to the PL of C_PE_-QDs^2,35^. As a matter of fact, surface-active groups and doping of C-QDs with heteroatoms play crucial factors that profoundly affect the photoluminescence. In particular, the electronegativity of heteroatoms (nitrogen/sulfur/Se) plays the role of multiple fluorescence centers^49^. For example, doping with S and Se, electron donors due to low electronegativity, fluorescence peak exhibits redshift. By contrast, doping with nitrogen, an electron acceptor, PL depicts the blue-shift. In the present study, the deconvolution of C_PE_-QDs XPS spectra of N 1*s* in Figure S7† shows two significant peaks at 399.03 (14.1 at.%) and 401.21 eV (85.9 at.%) corresponds to the Pyridinic-N and Graphitic-N, respectively (Table S4†) that predominantly provide a plausible explanation for the existence of blue-shift. Therefore, the excitation-dependent PL in C_PE_-QDs can be ascribed to the synergistic effects of quantum confinement, functional groups, and doping of heteroatoms^46,47,49^. It is worth noting, C_PE_-QDs had narrower redshift (40 nm), mainly because of the narrow size distribution of C_PE_-QDs than those synthesized by other methods such as electrochemical (70 nm), hydrothermal method (100 nm) and microwave pyrolysis (150 nm)^8,50,51^. Notably, the nitrogen doping minimizes the O-states and assist in enhancing the N-states in C-QDs, leading to the smaller PL shift^46^.

Recent studies showed that the PL in carbon dots (CDs) was originated from the fluorophores, and we have explored the possibility of the formation of fluorophores. Recent study showed that if the fluorophores exist, CD’s intrinsic structure, with little or no-graphitic formation, contributes to the PL emission of CDs^52–55^. However, in the present report, the chances that the as-synthesized C_PE_-QDs have fluorophores are minimal. Concerning the fluorophores, there are two possibilities; either fluorophores are present independently in addition to the CDs^54^ or attached on the surface of CDs^52^. Recent reports revealed that the fluorophores-functionalized CDs had rather a high quantum yield (QY) "usually more than 50.0%". On the contrary, the CDs alone (absence of fluorophores) had far lower QY “around 7.0%”. In the present report, as synthesized C_PE_-QDs has a quantum yield of ~ 5.0%, the QY of our C_PE_-QDs is aligned with^52^, where the CDs alone, absence of fluorophores, had significantly lower QY in comparison to fluorophores-functionalized CDs. Therefore, the possibility that the as-synthesized C_PE_-QDs have fluorophores attached to the surface of C-QDs was excluded. Furthermore, the formation of fluorophores is required the mild reaction conditions; short-reaction time and low temperature 150 ~ 200 °^ͦ^C, and if fluorophores are present, the hydrodynamic diameter of fluorophores is around ~ 1 nm.^54^ Given the fact that after high-energy electrons of plasma strikes on to the specimen, the instant temperature in localized-zone might even reach higher than 800 °C. The chances of forming the fluorophores are less likely than being carbonized. TLC results of dialyzed C_PE_-QDs exhibit the presence of a single fraction (Figure S11†). Also, the DLS measurement of C_PE_-QDs indicates “no peak” having a hydrodynamic-diameter around 1.0 nm. These observations successfully validate the assumption that fluorophores do not exist either independently for attached to the surface of C_PE_-QDs.^54^ Single fraction in TLC results illustrates that C_PE_-QDs have only one species and thus indicated the high purity of C_PE_-QDs^56^.

The photoluminescence (PL) of the C-QDs is sensitive to the external factors such as the pH and dispersant^36^. Figure 8C shows the PL of C_PE_-QDs dispersed in the water at excitation 320 nm for different pH ranging from 5 to 13 (acidic to basic). Irrespective of the excitation, the PL of C_PE_-QDs has a broad emission peak position, centered at 412 nm. Changing the pH from extreme acidic to basic, at first, resulting in a gradual increase in the PL intensity from pH 5 to pH 7. Subsequently, the PL intensity gradually decreases from pH 9 to pH 13. The highest PL intensity of C_PE_-QDs was found to be at the neural condition, i.e., pH 7. As the pH changes, the protonation–deprotonation and the existence of surface trap states on the surface of C_PE_-QDs might be the instrumental factor for the quenching of PL intensity. Noting, the surface trap states include dangling bonds, functional groups, and *sp*^2^–*sp*^3^ hybridized carbon atoms in the C_PE_-QDs. In particular, the PL emission peak of C_PE_-QDs shifts marginally towards higher wavelength (i.e., 2.4 nm) in strong acidic (pH = 5) to basic media (pH = 13). This marginal shift signifies that the PL peaks are almost independent of pH at fixed excitation energy (Fig. 8D)^36^. It is a well-known fact that the multiple surface-states dominate the PL in CDs. For instance, the different set of surface states gets activated, resulting in the redshift in PL. Whereas, PL of as-synthesized C_PE_-QDs in the present report, was found to be independent as the pH changes from 5 to 13, despite having a moderate functionalization. Therefore, it can be inferred that functionalization is not contributing to the PL of C_PE_-QDs, and functionalization only assists in achieving better colloidal stability.

In the section that follows, a thorough discussion focusing primarily, both the C-QDs synthesis process is elaborated; MPED and the conventional thermal decomposition. Unlike the C_PE_-QDs, C_PY_-QDs have a slightly broad diameter distribution in the range of 2.5–6.6 nm, i.e., average nanoparticle diameter 4.28 ± 0.91 nm, despite using the same carbon source as the starting materials (Figure S12†). It is worth noting that TEM images show that the C_PY_-QDs have various shapes; triangular/pentagon/spherical (Figure S13†), whereas the C_PE_-QDs were spherical (Fig. 4) and had a narrow size distribution in the range of 3.1–6.0 nm. The narrow size distribution of C_PE_-QDs, in the present report, is comparable to the C-QDs synthesized with the hydrothermal method using the synthetic carbon precursors.^27^ Remarkably, such kind of narrow size distribution is difficult to achieve using the natural carbon source^15,35^. Although, there are multiple reports where the authors have used the pyrolysis method and natural carbon source as starting materials; nevertheless, the synthesized C-QDs had a considerable size variation (Table S4†)^36^.

The PL of C_PY_-QDs dispersed in water at various excitations; 260, 280, 300, 320, 340, 360, and 380 nm, respectively (Figure S14†). The maximum emission intensity of C_PY_-QDs was recorded at excitation 260 nm. The peak fitting indicates that the shape of typical C_PY_-QDs PL emission spectra is an ensemble of two peaks; (1) peak centered at 436 nm (FWHM 57 nm), and (2) another peak at 470 nm (FWHM 101 nm). Although, the shape of PL spectra of C_PY_-QDs and C_PE_-QDs was very much similar, nevertheless, the PL peak width of C_PY_-QDs is marginally wider than C_PE_-QDs; it might be due to the broader size distribution of C_PY_-QDs compare to the C_PE_-QDs and/or presence of rich O-states in C_PY_-QDs than C_PE_-QDs. Quantitative XPS analysis of C_PY_-QDs validate the fact that C_PY_-QDs have rich O-states; XPS of survey scan, C 1*s*, O 1*s*, and N 1*s* are summarized in Tables S9†, S10†, S11†, and S12†, respectively. Also, the deconvolution of C 1*s*, O 1*s*, and N 1*s* is shown in Figure S8D†, S8E†, and S8F†, respectively. Although, the deconvoluted spectra of C 1*s*, O 1*s* and N 1*s* of C_PY_-QDs is very much similar to the C_PE_-QDs. Nevertheless, C_PY_-QDs XPS has three noticeable differences compared with C_PE_-QDs; (1) C_PY_-QDs have higher oxygen states (i.e., 40.2 at.%), (2) C_PY_-QDs have lower nitrogen states (i.e., 1.6 at.%), and (3) lower amount of Graphitic-N states (i.e., 60.0 at.%).

Actually, the PL emission spectra of C_PY_-QDs were nearly independent of the excitation wavelength. Figure S15† illustrate the comparison between C_PE_-QDs and C_PY_-QDs PL spectra, C_PY_-QDs PL shows a smaller redshift of 16 nm in comparison to the C_PE_-QDs (redshift of 40 nm). C_PY_-QDs had a broader diameter distribution. One would expect the more significant amount of redshift in C_PY_-QDs than the C_PE_-QDs, and quantum confinement could have been the plausible explanation. However, such kind of possibility was ruled out because C_PY_-QDs owning wider size distribution shows small redshift. We believe that the multifaceted shape of C_PY_-QDs (Figure S13†), presence of rich O-states in C_PY_-QDs (Figure S16†), and a lower amount of nitrogen states (i.e., 1.6 at.%) compared to the C_PE_-QDs (i.e., 5.6 at.%) might be the cause of the nearly-independent (i.e., smaller redshift) PL transitions in C_PY_-QDs^46^. Several reports show excitation-independent PL mode can be achieved by altering the surfaces, shapes, or carbonization degree of C-QDs^47,49,57^. The photoluminescence of C_PY_-QDs and C_PE_-QDs dispersed in the water at different pH ranging from 5 to 13 (Figure S17†) were found to be independent of the pH.

The quantum yield of C_PE_-QDs was comparable with C_PY_-QDs. For instance, the quantum yield of as-synthesized C_PE_-QDs (excitation wavelength 340 nm) and C_PY_-QDs (excitation wavelength 260 nm) was calculated to be 4.96 and 4.44%, respectively.^12,39^ Essentially, we would like to emphasize that the synthesis of C_PE_-QDs was 97.2% faster than the C_PY_-QDs process. Indeed, C-QDs synthesized from natural carbon sources have a lower quantum yield than synthetic carbon precursors^58,59^. However, the objective was to demonstrate the efficacy of the MPED process. It is believed that the quantum yield of C-QDs synthesized by MPED using the synthetic carbon precursors might even reach higher than natural carbon sources, especially if the C-QDs are made with synthetic carbon precursors.

To explore further the benefits of C-QDs synthesis using the MPED over the conventional process, the pros and cons are discussed in detail. In the process of C-QDs synthesis, scientists are trying to achieve a short reaction time that, in turn, would minimize the energy requirement. As a consequence, the carbon emission footprint could be significantly minimized. Chemical techniques, such as thermal decomposition, hydrothermal, solvothermal, autoclave, and electrochemical synthesis, require higher energy and prolonged reaction time^8,17,60,61^. C-QDs synthesis by hydrothermal requires temperature 180 °C for 6 h, the solvothermal method needs reaction temperature (> 150 °C) for 0.5 h, and much higher temperature for autoclave up to 200 °C for 2.5–12 h^59,60^. Although thermal activation speeds up the chemical reactions but requires enormous energy expenditures that lead to an undesired carbon emission footprint; consequently, the open question is whether such an energy-consuming process should be carried out on an enlarged scale. In practice, there are other intuitive electric/electromagnetic techniques (EMT); microwave-aided^62^, microwave oven^63^, laser ablation^4^, and arc discharge synthesis^3^, such EMT is faster, and energy-efficient. When the microwave is employed, due to dielectric heating, the irradiation time decreases significantly (1–30 min), microwave-assisted synthesis process required 800 W for 20 min, and microwave oven 800 W for 3 min^62^. Similarly, with laser ablation, the time required for the C-QDs synthesis is much shorter, i.e., less than 90 s^4^. Nevertheless, some drawback remains for above techniques, such as; low quantum yield, poor crystallinity, short reaction time is only applicable to the synthetic carbon precursors, reaction time is as long as the hydrothermal process and challenges to rapidly convert highly concentrated carbon precursors into C-QDs without any assistance^52,64,65^.

To overcome the challenges discussed in the preceding section, the purposed MPED process is a preferred alternative. MPED is an ultra-fast, low cost, and highly energy-efficient process. MPED method is universal; i.e., various kinds of dry seeds, plant leaves, and synthetic protein powder were employed to synthesize C-QDs. We have also successfully synthesized the C-QDs from the mixture of the natural and synthetic carbon source via MPED. The distinct benefit of MPED was the energetic plasma and plasma associated radicals injected directly into the reaction volume, as a consequence, maximized the atom economy and curtail the energy required for the synthesis of C-QDs. MPED process is very intuitive because verities of the natural carbon sources can be screened quickly; this process can be particularly beneficial for those natural carbon sources that have lower flashpoint or/and water solubility. Furthermore, using such carbon sources, the necessity of the external temperature is avoidable. The amount of C-QDs produced by the MPED process is approx. 27% higher than the conventional pyrolysis synthesis process. Finally, yet importantly, to the best of our knowledge, using the natural carbon precursor, the fastest and highly energy-efficient C-QDs synthesis process required at least 15 min and 7.2 × 10^5^ J of energy (microwave oven). Alternatively, our proposed MPED process is three-times faster and consumes far less energy (1.5 × 10^5^ J) than the microwave oven method^66^.

### Application of C-QDs: florescent lysozyme protein crystals

**Figure 9:**
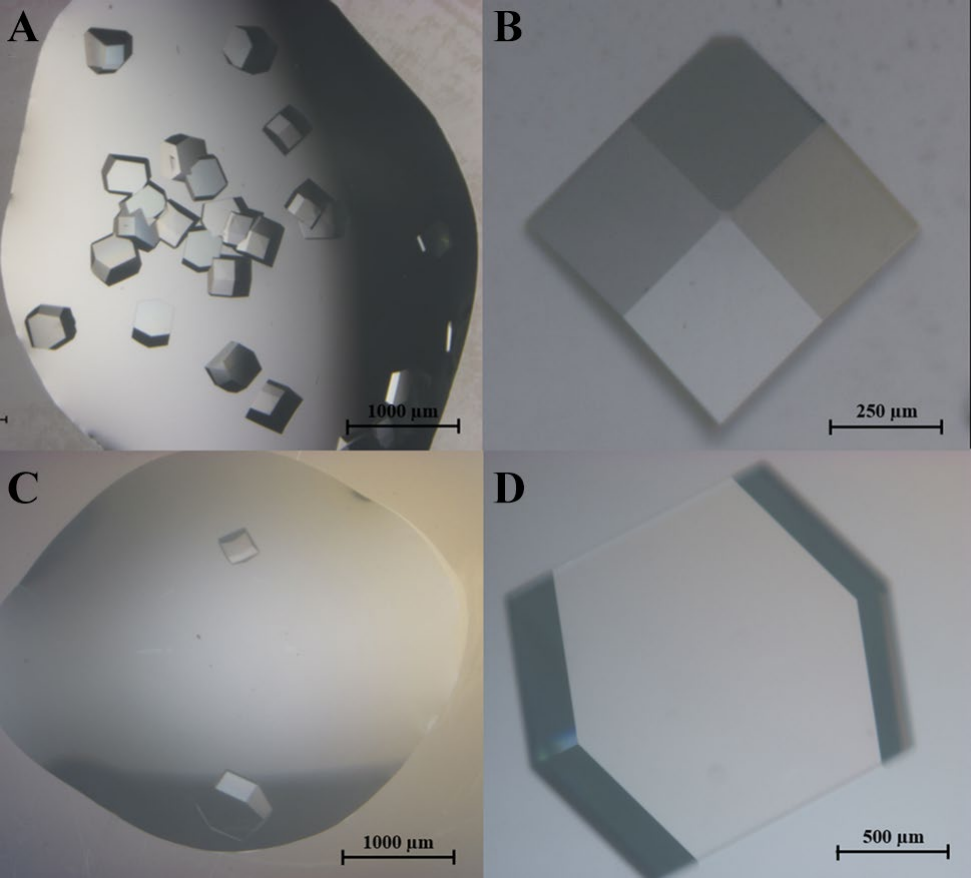
(**A**) Optical images of as-grown Lysozyme at low resolution that shows high nucleation of crystals (scale bar 1,000 μm), (**B**) Optical images of as-grown Lysozyme at high resolution (scale bar 250 μm), (**C**) Optical images of CQD-Lysozyme protein crystal at low resolution that shows lower nucleation of crystals (scale bar 1,000 μm), (**D**) Optical images of CQD-Lysozyme protein crystal at high resolution (scale bar 500 μm).

Figure 9 shows the optical images of as-grown Lysozyme and CQD-Lysozyme protein crystals. Both the crystals, Lysozyme and CQD-Lysozyme, were completely transparent and possessed tetragonal prismatic morphology. Optical images show no sign of any clumps irrespective of the crystals. Even after the C-QDs were incorporated, the CQD-Lysozyme protein crystal retained the characteristic features of tetragonal prismatic morphology. Noting, given the fact that on many occasions, such foreign NMs 'guest' was precipitated and found to be lying alongside the host matrix of bio-Nano systems^28,29^^.^ For lysozyme protein crystals, the average number of crystals and size was found to be ~ 30/droplet and 300–400 μm, respectively. On the contrary, CQD-Lysozyme crystals were larger in size and fewer/droplet, the number of as-grown crystals/droplet were in the range of 3 to 8 crystal, the average number of crystals and size were found to be ~ 3/droplet and 500–700 μm, respectively. It is evident from the results that CQD-lysozyme crystals were larger in size, and the nucleation rate was almost ten times slower than Lysozyme protein crystals.

**Figure 10:**
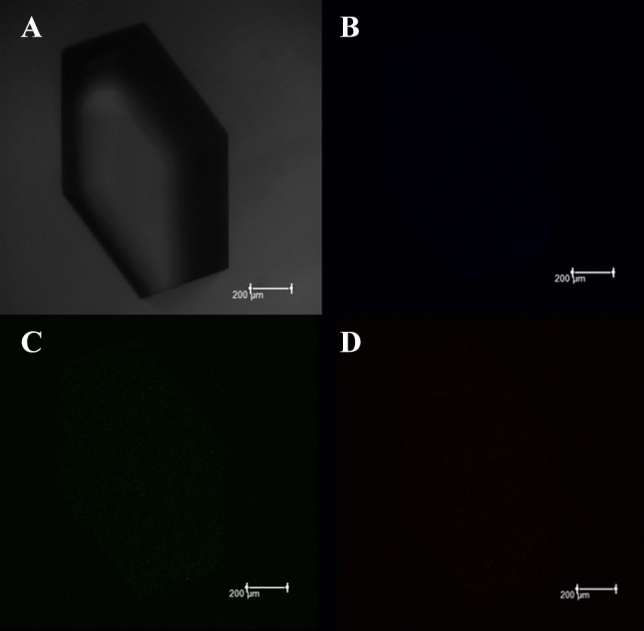
Confocal images of Lysozyme protein crystal shows (**A**) Optical image of Lysozyme protein crystal, (**B**) Lysozyme protein crystal emit slight fluorescence only in the blue region, (**C**) Fluorescence signal was absent at the green excitation wavelength in lysozyme protein crystal, (**D**) Fluorescence signal was absent at the green excitation wavelength in lysozyme protein crystal.

**Figure 11:**
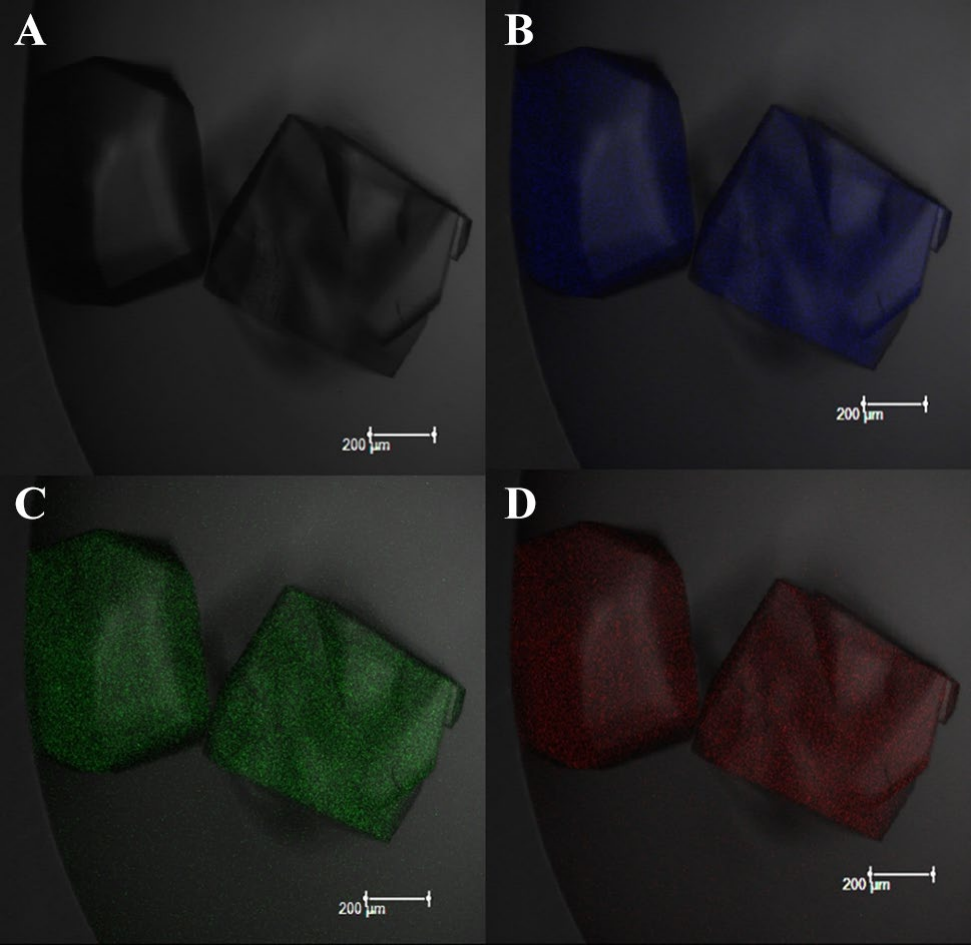
Confocal images of CQD-Lysozyme protein crystal show (**A**) Optical image of CQD-Lysozyme protein crystal, (**B**) CQD-Lysozyme protein crystal emit fluorescence in the blue region, (**C**) CQD-Lysozyme protein crystal emit fluorescence in the green region, (**D**) CQD-Lysozyme protein crystal emit fluorescence in the red region.

Confocal fluorescence microscopy was utilized to examine the fluorescence properties of both kinds of crystals. Figure 10 shows the typical behavior of as grown Lysozyme protein crystal, lysozyme being a protein is known to emit slight fluorescence only in the blue region. The fluorescence signal was absent at the green and red excitation wavelength, respectively. Conversely, Fig. 11 shows that the CQD-Lysozyme protein crystals have the tunable luminescence, blue, green, and red fluorescence emissions were observed when the samples were excited at 410–490 (blue), 515–560 (green), 610–620 nm (red), respectively. Uniform distributions of fluorescence intensity were observed throughout the as grown C-Lysozyme protein crystal, indicating that the C-QDs were possibly homogeneously dispersed within the C-Lysozyme protein.

Organic and inorganic quantum dots were synthesized in-situ into the lysozyme protein crystals^67,68^. However, the present report, as per our knowledge, is the first to demonstrate that ex-situ incorporation of C-QDs into the lysozyme protein crystals. Although, the fluorescence of C-QDs often gets quenched quite easily, especially on occasion such as casting the film or dispersed into the other materials^23^. On the contrary, the intrinsic fluorescence of the C-QDs was preserved into the Lysozyme protein matrix; fluorescence was attributed primarily to the presence of C-QDs. Optical and confocal images show that lysozyme protein is a favorable host matrix for the “guest” C-QDs. Unlike the complicated procedure reported in the past, our preparation method is straight forward and does not involve the cumbersome arrangements and harsh synthesis chemicals^23,67,69^. Such kind of CQD-Lysozyme protein crystals could provide a platform for the development of next-generation polychrome luminescent crystals.

## Conclusion

In the present study, we demonstrated the rapid synthesis of mono-dispersed C-QDs using MPED; highly crystalline C-QDs were synthesized in a matter of 5 min. Synthesis of C-QDs via MPED is a single-step, energy-efficient, and highly reproducible process that requires no external temperature. We emphasize that hydrogen (H_2_) plasma containing the high-energy electrons and activated hydrogen ions predominantly provide the required energy, upon striking on to the ground fenugreek powder, thus maximizing the atom economy. Intrinsically N-doped C_PE_-QDs provide an opportunity to tune the fluorescence in dual-mode, blue, and redshift with selective heteroatoms doping. The excitation dependent PL in C_PE_-QDs was ascribed to the synergistic effects of quantum confinement, functional groups, and doping of heteroatoms. Necessarily, we would like to emphasize that C-QDs synthesized with MPED have narrow diameter distribution, and the process was 97.2% faster than conventional thermal decomposition, despite having the very similar Quantum yield. Lysozyme protein is a potential host to produce C-QDs based florescent lysozyme protein crystals. Such a guest–host strategy would encourage the development of diverse and complex “bio-nano systems”.

## Supplementary information

Supplementary Information 1.
