# Supplementary material for: Ultrafast synthesis of carbon quantum dots from fenugreek seeds using microwave plasma enhanced decomposition: application of C-QDs to grow fluorescent protein crystals
Source: Sci Rep. 2020 Jul 23;10:12333. doi: 10.1038/s41598-020-69264-9 (PMC7378176; doi:10.1038/s41598-020-69264-9)
Supplement: Supplementary file 1 — Supplementary Information 1. [file 41598_2020_69264_MOESM1_ESM.pdf]

Supporting Information

Ultrafast Synthesis of Carbon Quantum Dots from Fenugreek Seeds using Microwave Plasma Enhanced Decomposition: Application of C-QDs to Grow Fluorescent Protein Crystals

Akansha Dager[1], Ankur Baliyan[2], Shunji Kurosu[3], Toru Maekawa[3], Masaru Tachibana[1]

[1]Graduate School of Nanobioscience, Yokohama City University, 22-2 Seto, Kanazawa-Ku, Yokohama 236-0027, Japan
[2]NISSAN ARC, LTD, 1-Natsushima-cho, Yokosuka, 236-0061, Japan
[3]Bio-Nano Electronics Research Centre, Toyo University 2100, Kujirai, Kawagoe, Saitama 350-8585, Japan

Contents

1. Synthesis of C_{PY}-QD using thermal decomposition.

2. Optical emission spectra (OES) of the excited hydrogen plasma.
Fig. S1

3. Distribution of the diameters of as synthesized C_{PE}-QDs via MPED.
Fig. S2

4. Higher resolution TEM image of C_{PE}-QDs synthesized via MPED.
Fig. S3

5. Point beam EDS spectra of as synthesized C_{PE}-QDs.
Fig. S4

6. Colloidal stability of as synthesized C_{PE}-QDs.
Fig. S5

7. XPS analysis of as-synthesized C_{PE}-QDs.
Fig. S6

8. Table: Quantitative Analysis of XPS analysis of as-synthesized C_{PE}-QDs.
Table S1- Wide scan of as-synthesized C_{PE}-QDs.
Table S2- Peak fitting results obtained after deconvolution of carbon peak.
Table S3- Peak fitting results obtained after deconvolution of oxygen peak.
Table S4- Peak fitting results obtained after deconvolution of nitrogen peak.

9. Deconvolution of nitrogen peak N 1s (XPS analysis) of as-synthesized C_{PE}-QDs.
Fig. S7

10. Table: Quantitative Analysis of XPS analysis of Fenugreek seeds.
Table S5- Wide scan of Fenugreek seeds.
Table S6- Peak fitting results obtained after deconvolution of carbon peak.
Table S7- Peak fitting results obtained after deconvolution of oxygen peak.
Table S8- Peak fitting results obtained after deconvolution of nitrogen peak.

11. Table: Quantitative Analysis of XPS analysis of as-synthesized C_{PY}-QDs.

Table S9- Wide scan of as-synthesized C_{PY}-QDs.

Table S10- Peak fitting results obtained after deconvolution of carbon peak.

Table S11- Peak fitting results obtained after deconvolution of oxygen peak.

Table S12- Peak fitting results obtained after deconvolution of nitrogen peak.

12. XPS analysis of Fenugreek-seeds and as-synthesized C_{PY}-QDs.

Fig. S8

13. Typical peak fitting of an individual PL emission spectrum of C_{PE}-QDs.

Fig. S9

14. Dual mode (blue & red shift) of excitation-dependent PL of C_{PE}-QDs.

Fig. S10

15. Thin layer chromatography of C_{PE}-QDs.

Fig. S11

16. Distribution of the diameters of as synthesized C_{PY}-QDs via thermal decomposition method.

Fig. S12

17. TEM images of multifaceted shape of C_{PY}-QDs synthesized by thermal decomposition method.

Fig. S13

18. PL emission spectra of C_{PY}-QDs excited at various energies.

Fig. S14

19. Comparison of PL spectra of as synthesized C_{PE}-QDs and C_{PY}-QDs.

Fig. S15

20. FTIR spectra of as synthesized C_{PY}-QDs.

Fig. S16

21. Effect of pH on PL of as synthesized C_{PY}-QDs (Environmental stability).

Fig. S17

22. Summery of the various synthesis techniques using natural carbon sources.

Table S13

23. References

1. Synthesis of C_{PY}-QD using thermal decomposition

Carbon Quantum dots were synthesized by thermal decomposition (pyrolysis) method. As received fenugreek seeds were crushed using a mixer grinder (Tiger mixer grinder, Japan). Ground fenugreek powder (0.2 g) was transferred to the crucible cup (AS ONE, Japan) and was heated using a heat plate (AS ONE, Japan) at a constant temperature of 500 °C for 3 hours. Subsequently, the crucible was allowed to cool down to room temperature. Carbonization of the fenugreek powder turned into a dark-gray product, and it was dissolved in deionized water followed by the sonication for 5 minutes. The black color suspension was centrifuged at 15000 rpm for 10 minutes to remove the large un-dissolved particles. The supernatant was filtered using 100 nm pore size filter (PALL ACRO DISC, Japan) and subjected to the dialysis using dialysis kit (Float-A-Lyzer G2 Dialysis, Japan) for further purification. The purified C_{PY}-QDs was transferred to the glass vial and stored for further characterization [1].

2. Optical emission spectra (OES) of the excited hydrogen plasma.

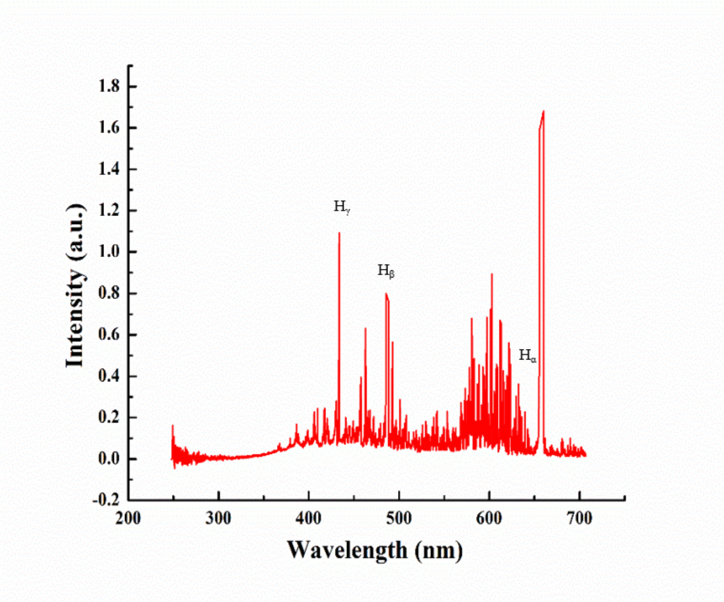

Fig. S1: The OES spectra show peaks at 658.2 486.9, 434.7 and 463.8nm that assigned to H_{α}, $H_{\beta,}$ H_{γ} and secondary hydrogen, respectively.

3. Distribution of the diameters of as synthesized C_{PE}-QDs via MPED.

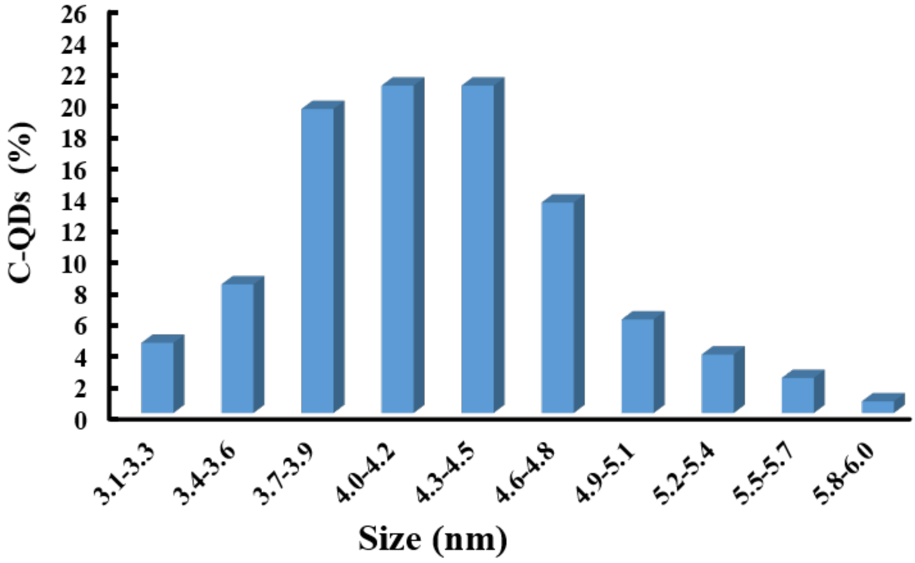

Fig. S2: Distribution of the diameters of as synthesized C_{PE}-QDs. The average diameter and standard deviation of C_{PE}-QDs were 4.25 and 0.56 nm, respectively. More than 90% of C_{PE}-QDs have diameter in the range of 3.1- 4.8 nm.

4. Higher resolution TEM image of C_{PE}-QDs synthesized via MPED.

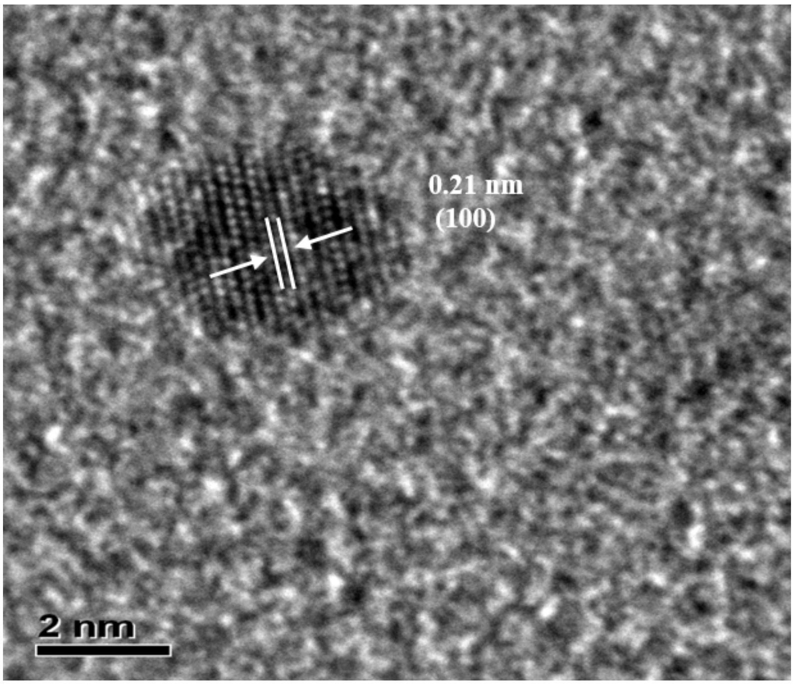

Fig. S3: Higher resolution TEM image of C_{PE}-QDs shows that C_{PE}-QDs have crystalline structure and the distance between the lattices fringes is 0.21 nm assigned to (100) plane.

5. Point beam EDS spectra of as synthesized C_{PE}-QDs.

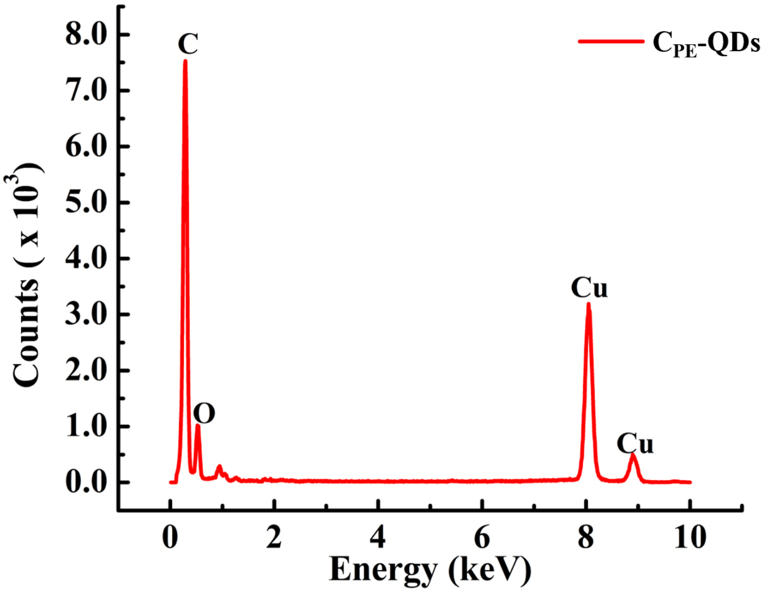

Fig. S4: Point beam EDS spectra of as synthesized C_{PE}-QDs. No other trace element was detected other than carbon and oxygen.

6. Colloidal stability of as synthesized C_{PE}-QDs.

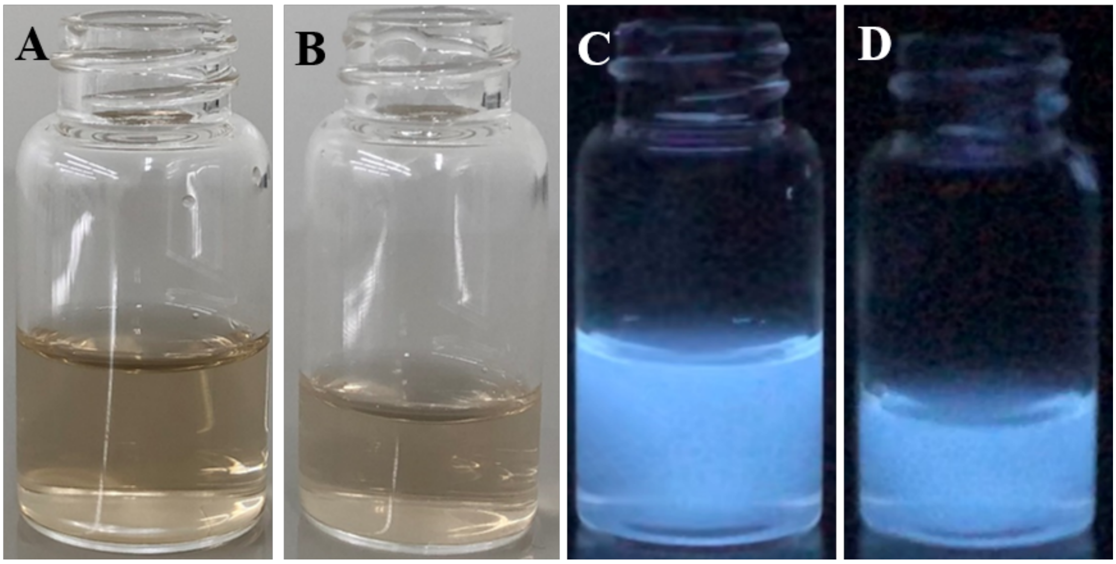

Fig. S5: C_{PE}-QDs shows no sign of turbidity even after 6 months of storage in ambient condition. (A) Optical image of Freshly synthesized C_{PE}-QDs dispersed in water under daylight exposure, (B) Optical image of C_{PE}-QDs dispersed in water under daylight exposure (after 6 months storage), (C) Optical image of Freshly synthesized C_{PE}-QDs dispersed in water under UV exposure, (D) Optical image of C_{PE}-QDs dispersed in water under UV exposure (after 6 months storage).

7. XPS analysis of as-synthesized C_{PE}-QDs.

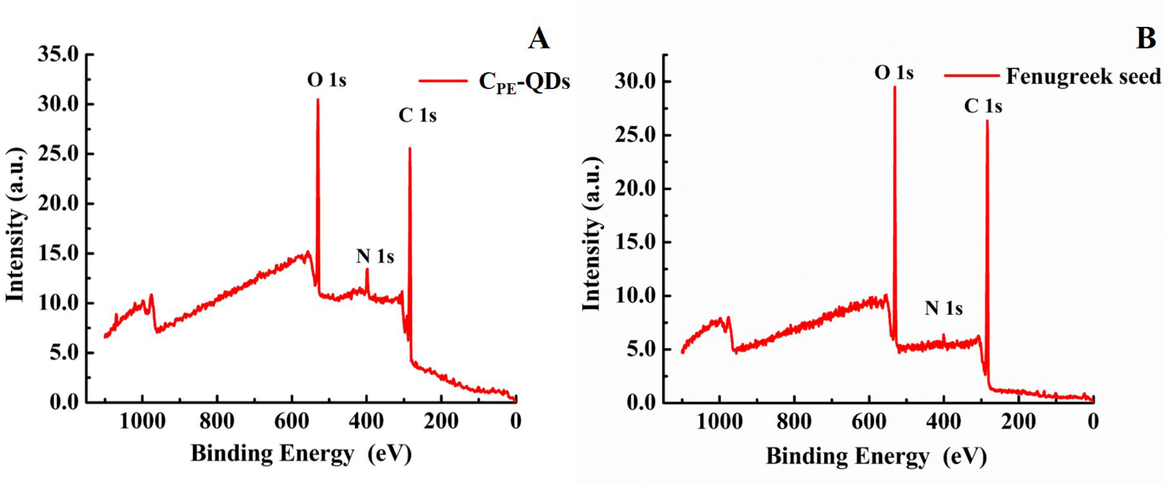

Fig. S6: XPS analysis of as-synthesized C_{PE}-QDs. (a) The survey scan of C_{PE}-QDs XPS shows that the carbon quantum dots are composed of mainly carbon, nitrogen and oxygen. (b) XPS survey scan of Fenugreek powder shows that the Fenugreek seeds were mainly composed of carbon, nitrogen and oxygen.

8. Table: Quantitative Analysis of XPS analysis of as-synthesized C_{PE}-QDs.

Table S1. XPS analysis of as-synthesized C_{PE}-QDs

S.No.	Element	Peak position BE (eV)	Atomic concentration (%)
1	C 1s	283.6	63.4
2	O 1s	531.0	31.0
3	N 1s	399.4	5.6

Table S2. Peak fitting results obtained after deconvolution of carbon peak

Peak	Assignment	Peak position BE (ev)	FWHM (eV)	Area (%)
C 1s	C-sp^2	283.60	1.27	77.5
C 1s	C-O	285.09	1.25	13.5
C 1s	C=O	286.79	1.33	8.9

Table S3. Peak fitting results obtained after deconvolution of oxygen peak

Peak	Assignment	Peak position BE (ev)	FWHM (eV)	Area (%)
O 1s	OH/C=O	529.58	1.82	65.9
O 1s	C-O	531.01	2.07	34.1

Table S4. Peak fitting results obtained after deconvolution of nitrogen peak

Peak	Assignment	Peak position BE (ev)	FWHM (eV)	Area (%)
N 1s	Pyridinic-N	399.03	1.35	14.1
N 1s	Graphitic-N	401.21	1.56	85.9

9. Deconvolution of nitrogen peak N 1s (XPS analysis) of as-synthesized C_{PE}-QDs.

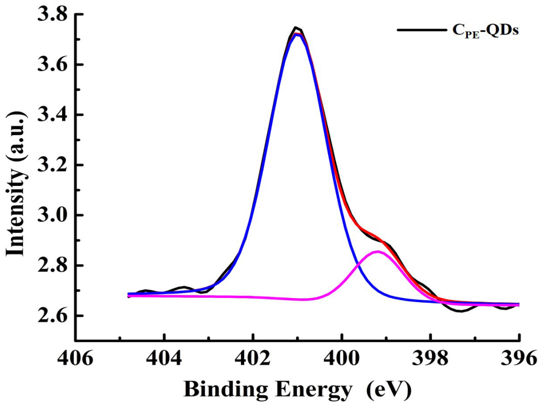

Fig. S7: XPS spectra of N 1s spectrum of as-synthesized C_{PE}-QDs. the spectrum was de-convoluted into two significant peaks at 399.03 (Pyridinic-N) and 401.21eV (Graphitic-N) respectively.

10. Table: Quantitative Analysis of XPS analysis of fenugreek seeds.

Table S5. XPS analysis of fenugreek seeds

S.No.	Element	Peak position BE (eV)	Atomic concentration (%)
1	C 1s	283.2	57.0
2	O 1s	531.0	40.4
3	N 1s	399.1	2.6

Table S6. Peak fitting results obtained after deconvolution of carbon peak

Peak	Assignment	Peak position BE (ev)	FWHM (eV)	Area (%)
C 1s	C-C sp^3	283.19	1.19	58.1
C 1s	C-H	284.81	1.47	34.9
C 1s	C-O	286.50	1.20	4.8
C 1s	C=O	287.75	1.02	2.2

Table S7. Peak fitting results obtained after deconvolution of oxygen peak

Peak	Assignment	Peak position BE (ev)	FWHM (eV)	Area (%)
O 1s	O-Adsorb	529.18	1.39	5.3
O 1s	C-O	531.04	1.81	94.7

Table S8. Peak fitting results obtained after deconvolution of nitrogen peak

Peak	Assignment	Peak position BE (ev)	FWHM (eV)	Area (%)
N 1s	$-NH_2$	397.87	1.37	20.4
N 1s	-C=N-R	398.94	1.30	19.6
N 1s	-C-N-R	400.99	1.99	60.0

11. Table: Quantitative Analysis of XPS analysis of as-synthesized C_{PY}-QDs.

Table S9. XPS analysis of as-synthesized C_{PY}-QDs

S.No.	Element	Peak position BE (eV)	Atomic concentration (%)
1	C 1s	283.6	58.2
2	O 1s	531.0	40.2
3	N 1s	399.4	1.6

Table S10. Peak fitting results obtained after deconvolution of carbon peak

Peak	Assignment	Peak position BE (ev)	FWHM (eV)	Area (%)
C 1s	C-sp^2	283.60	1.49	68.5
C 1s	C-O	284.92	1.61	24.5
C 1s	C=O	286.80	1.19	7.0

Table S11. Peak fitting results obtained after deconvolution of oxygen peak

Peak	Assignment	Peak position BE (ev)	FWHM (eV)	Area (%)
O 1s	OH/C=O	529.53	1.71	56.4
O 1s	C-O	530.91	1.98	43.6

Table S12. Peak fitting results obtained after deconvolution of nitrogen peak

Peak	Assignment	Peak position BE (ev)	FWHM (eV)	Area (%)
N 1s	Pyridinic-N	398.54	1.52	24.4
N 1s	Pyrrolic-N	400.30	1.11	14.8
N 1s	Graphitic-N	401.52	2.03	60.7

12. XPS analysis of Fenugreek-seeds and as-synthesized C_{PY}-QDs.

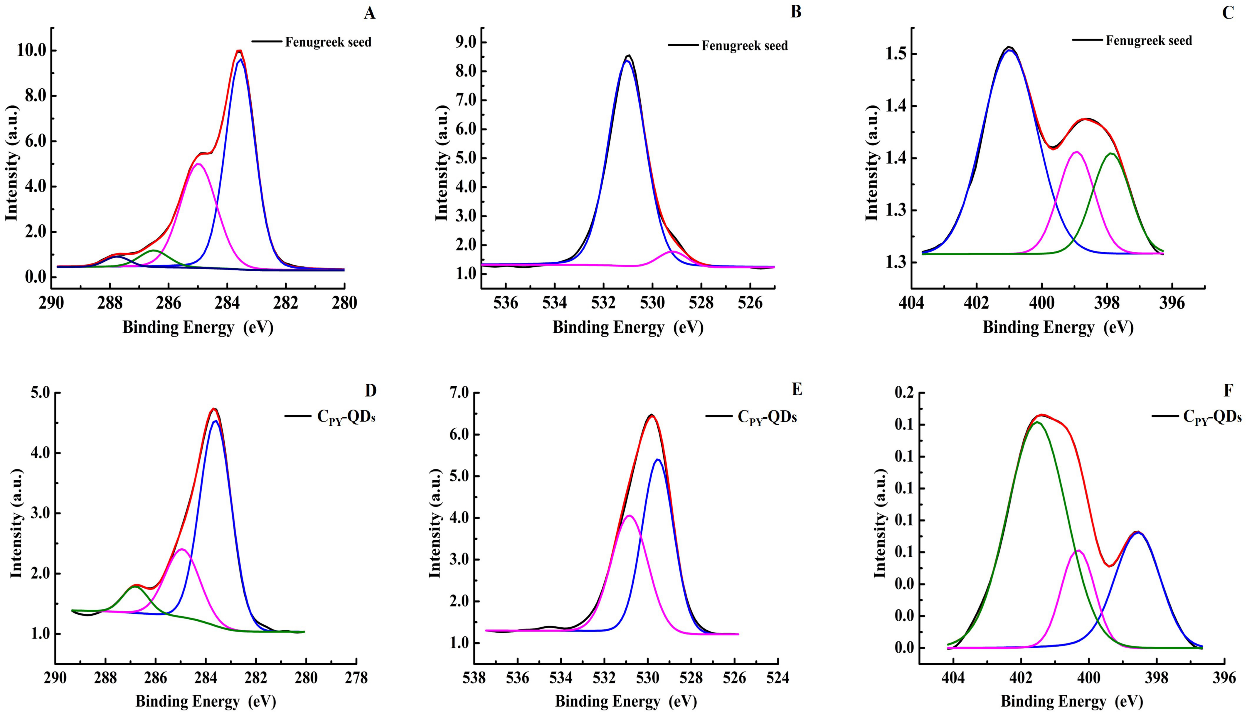

Fig. S8: XPS analysis of Fenugreek-seeds and as-synthesized C_{PY}-QDs. **(A)** XPS spectra of C 1s spectrum of fenugreek-seeds, the spectrum was de-convoluted into four significant peaks at 283.19 (C-C sp^3), 285.09 (C-H), 286.79 (C-O), and 286.79eV (C=O), respectively, **(B)** XPS spectra of O 1s spectrum of fenugreek-seeds, the spectrum was de-convoluted into two significant peaks at 529.18 (O-Adsorb), and 531.04eV (C-O), respectively, **(C)** XPS spectra of N 1s spectrum of fenugreek-seeds, the spectrum was de-convoluted into three significant peaks at 397.87 ($-NH_2$), 398.94 (-C=N-R) and 400.99eV (-C-N-R), respectively, **(D)** XPS spectra of C 1s spectrum of as-synthesized C_{PY}-QDs, the spectrum was de-convoluted into three significant peaks at 283.60 (C-sp^2), 284.92 (C-O) and 286.80eV (C=O) respectively, **(E)** XPS spectra of O 1s spectrum of as-synthesized C_{PY}-QDs, the spectrum was de-convoluted into two significant peaks at 529.53 (OH/C=O) and 530.91 (C-O) respectively, **(F)** XPS spectra of N 1s spectrum of as-synthesized C_{PY}-QDs, the spectrum was de-convoluted into three significant peaks at 398.54 (Pyridinic-N), 400.30 (Pyrrolic-N) and 401.52eV (Graphitic-N) respectively.

13. Typical peak fitting of an individual PL emission spectrum of C_{PE}-QDs

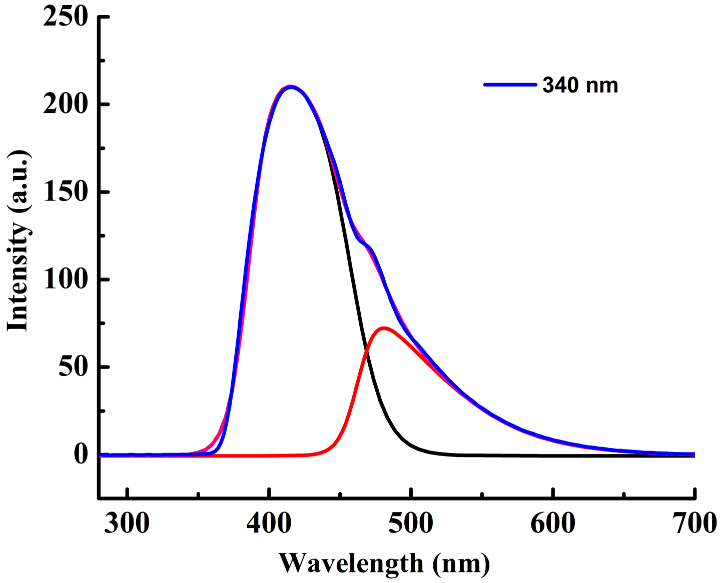

Fig. S9: Typical PL emission spectra is composed of two peaks; (i) the main peak centered at 414 nm (FWHM 46 nm), and (ii) another peak at 468 nm (FWHM 88 nm)

14. Dual mode (blue & red shift) of excitation-dependent PL of C_{PE}-QDs.

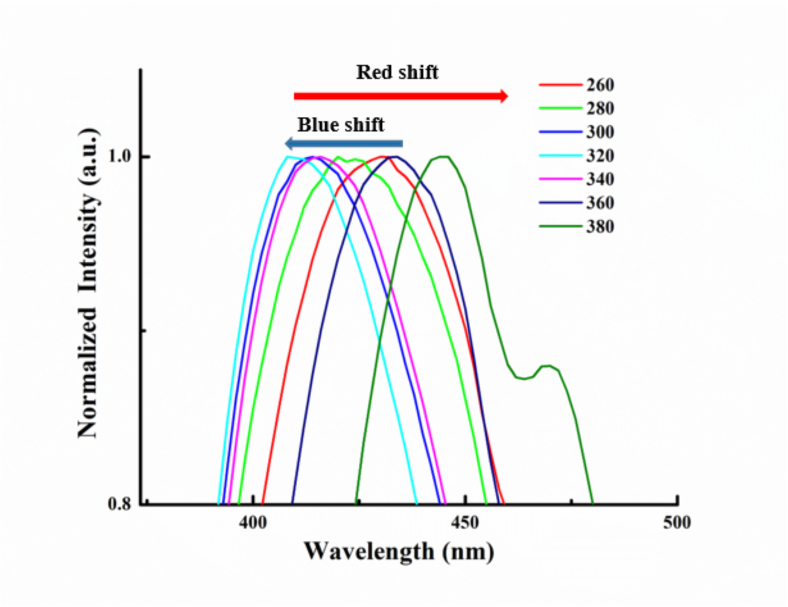

Fig. S10: Dual mode (blue & red shift) of C_{PE}-QDs. Blue-shift (22 nm) as the excitation wavelength changes from 260 - 320 nm and redshift (40 nm) when C_{PE}-QDs were excited with 340 - 380 nm; the total shift (blue + red) was found to be 62 nm

15. Thin Layer Chromatography of C_{PE}-QDs.

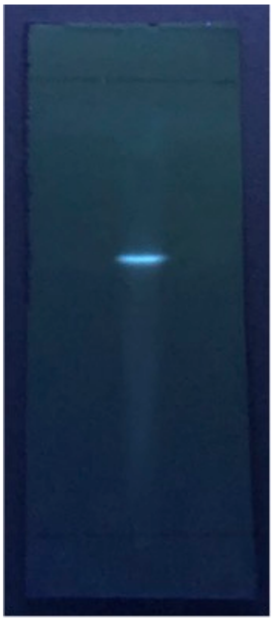

Fig. S11. Thin layer chromatography (TLC) of as synthesized C_{PE}-QDs. TLC shows a single luminescent band.

16. Distribution of the diameters of as synthesized C_{PY}-QDs via thermal decomposition method.

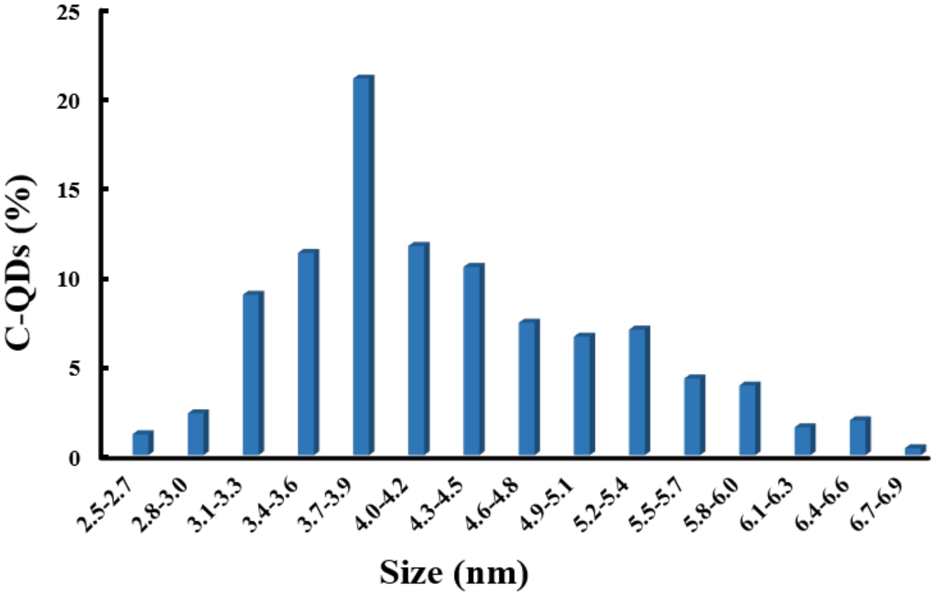

Fig. S12: Distribution of the diameters of as synthesized C_{PY}-QDs. C_{PY}-QDs have diameter distribution in the range of 2.5 - 6.6 nm, i.e., average nanoparticle diameter 4.28 ± 0.91 nm.

17. TEM images of multifaceted shape of C_{PY}-QDs synthesized by thermal decomposition method.

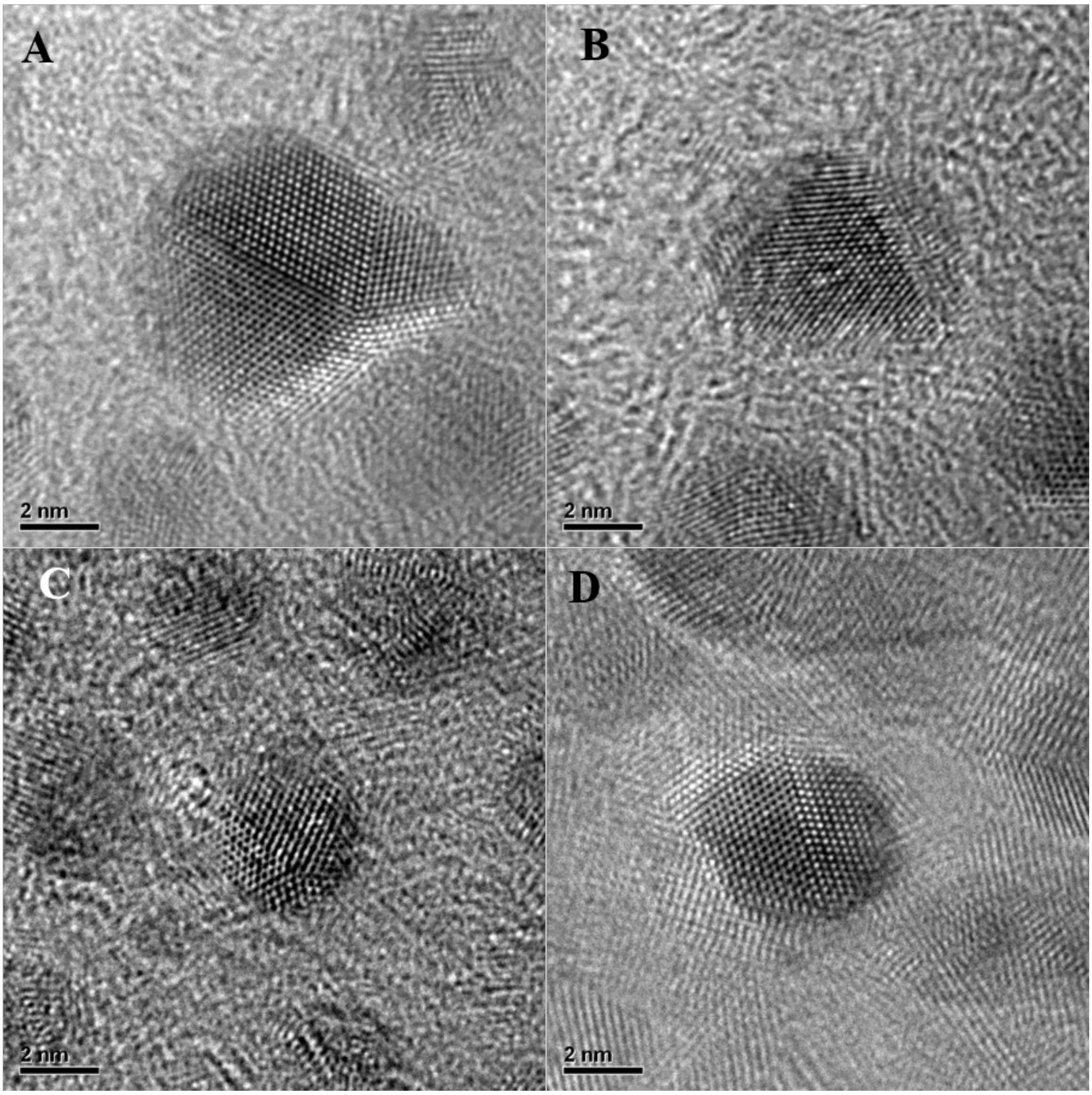

Fig. S13: TEM images show that the C_{PY}-QDs have various type of shapes; triangular/pentagon/ spherical. (a) Pyramidal. (b) Triangle. (c) Spherical/ Pyramidal. (d) Pentagon.

18. PL emission spectra of C_{PY}-QDs excited at various energies.

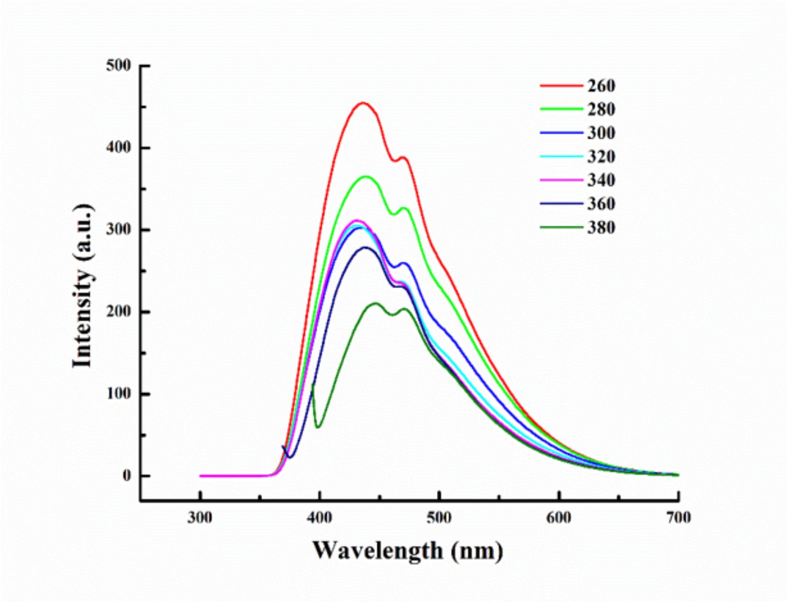

Fig. S14: PL emission spectra of C_{PY}-QDs excited at various energies (260, 280, 300, 320, 340, 360 and 380nm, C_{PY}-QDs were found to be nearly independent on the excitation energy.

19. Comparison of PL spectra of as synthesized C_{PE}-QDs and C_{PY}-QDs.

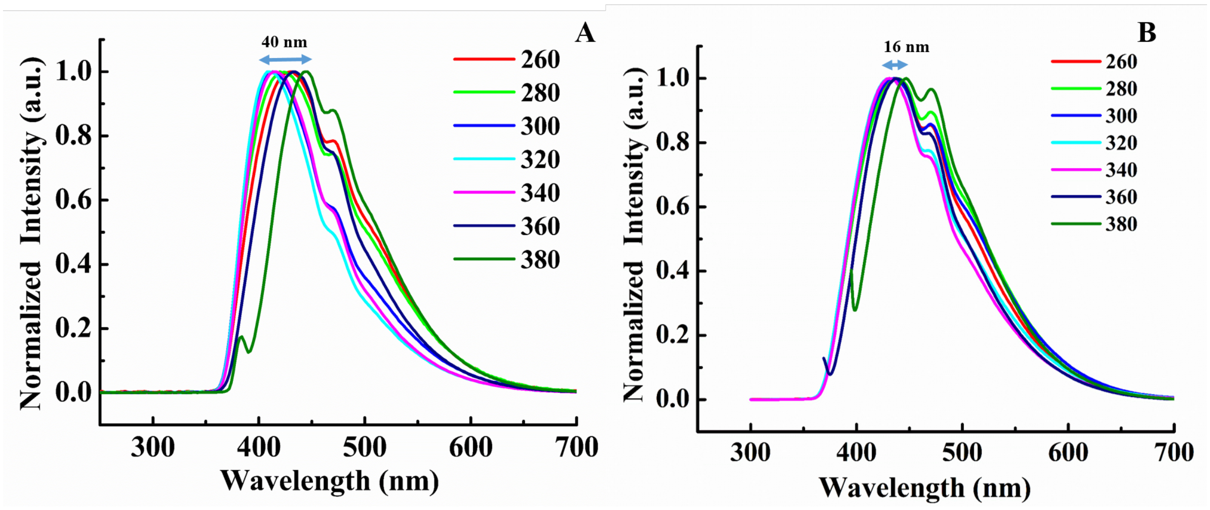

Fig. S15: (A) PL emission spectra of as synthesized C_{PE}-QDs, (B) PL emission spectra of as synthesized of C_{PY}-QDs. As synthesized C_{PY}-QDs show a smaller red-shift of 16 nm compared to the C_{PE}-QDs (red-shift of 40 nm).

20. FTIR spectra of as synthesized C_{PY}-QDs

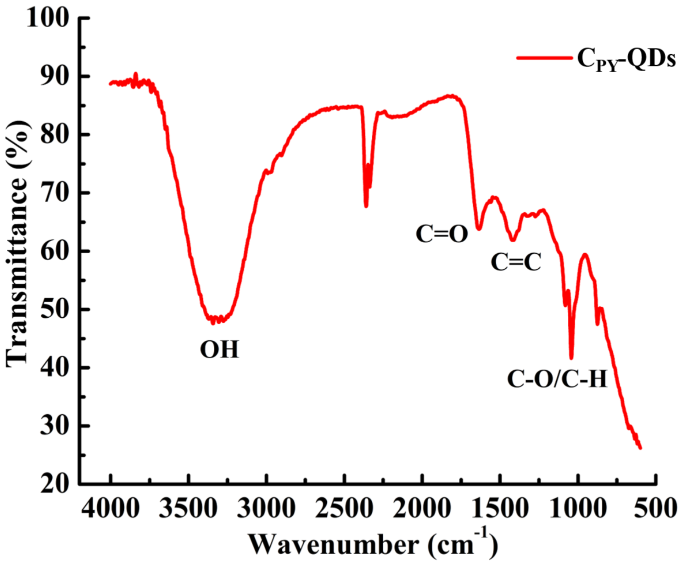

Fig. S16: FTIR spectra of as-synthesized C_{PY}-QDs, Carbon quantum dots have mainly the C=O, OH and C-O peaks, respectively.

21. Effect of pH on PL of as synthesized C_{PY}-QDs (Environmental stability)

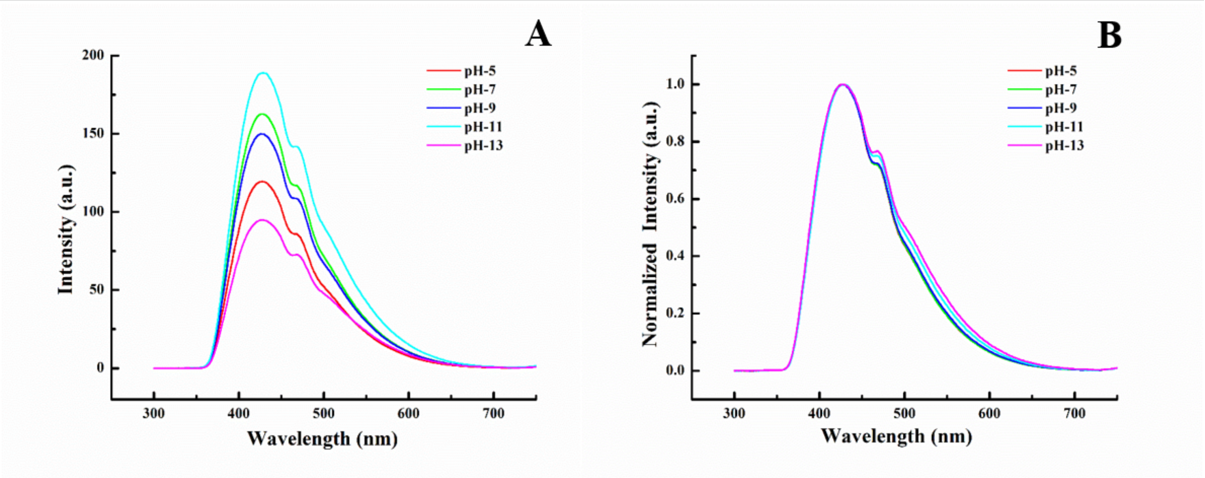

Fig. S17: PL emission spectra of C_{PY}-QDs dispersed in water at various pH (acidic to basic) (excitation wavelength 320 nm). C_{PY}-QDs were found to be independent of the excitation energy.

22. Summery of the various synthesis techniques using natural carbon sources

Table S13. C-QDs synthesis using various synthesis techniques

S.No.	Natural Carbon Source	Sustainable (Yes/No)	Additive Required (Yes/No)	Solvent Required (Yes/No)	Size of C-QDs (nm)	PL excitation dependent (Yes/No)	Quality of C-QDs-Graphitized (Yes/No)	Reaction Conditions (Temp, time)	Method	Ref.
1.	Watermelon peel	No	-	Water	1.5-4.0	Dependent	No	220 °C, 2 h	Carbonization	2
2.	Hair	No	-	Water	2-8	Dependent	No	200 °C, 24h	Autoclave	3
3.	Lemon juice	No	-	Water	2.0-30	Dependent	No	150 °C, 40min	Carbonization	4
4.	Chitosan	Yes		Water	1.0-6.1	Dependent	yes	300 °C, 2 h	Carbonization	5
5.	Cashew gum	Yes	-	Water	5.0 - 9.0	Dependent	No	800 W, 40 minutes	Microwave	6
6.	Orange Juice	No	-	Ethanol	1.5-4.5	Dependent	No	120 °C, 2.5 h	Hydrothermal	7
7.	Carrot juice	No	-	Water	3.0-8.0	Dependent	No	160°C, 6h	Hydrothermal	8
8.	Cabbage	No	-	Water	2.0-6.0	Dependent	No	140 °C, 5 h	Hydrothermal	9
9.	Papaya	No	-	Ethanol	8.0-10	Dependent	No	200 °C, 5 h	Hydrothermal	10
10.	Sweet potato	No	-	Water	1.1-5.6	Dependent	No	180 °C, 18 h	Hydrothermal	11
**11.**	**Fenugreek Seeds**	**Yes**	**No**	**No**	**3.1-5.8**	**Dependent**	**Yes**	**No external temperature, 5 min**	**MPED**	

23. Reference:

1. A. Dager, T. Uchida, T. Maekawa, M. Tachibana *Sci Rep*., **9**, 14004 (2019).
2. J. Zhou, Z. Sheng, H. Han, M. Zou, C. Li, *Materials Letters,* 2012, **66**, 222–224.
3. Y. Guo, L. Zhang, F. Cao & Y. Leng, *Sci Rep.*, 2016, **6**, 35795.
4. E. M. Schneider, A. Bartsch, W. J. Stark, and R. N. Grass, *J. Chem. Educ.* 2019, **96**, 540−545.
5. X. Liu, J. Pang, F. Xu and X. Zhang, *Sci Rep.*, 2016, **6**, 31100.
6. N. R. Pires, C. M. W. Santos, R. R. Sousa, R. C. M. d. Paula, P. L. R. Cunha and J. P. A. Feitosa, *J. Braz. Chem. Soc.,* 2015, **26**, 1274-1282.
7. S. Sahu, B. Behera, T. K. Maiti and S. Mohapatr, *Chem. Commun*., 2012, **48**, 8835–8837
8. Y. Liu, Y. Liu, M. Park, S. J. Park, Y Zhang, M. R. Akanda, B. Y. Park and H. Y. Kim, Carbon., 2017, **21**, 61-67.
9. A.M. Alam, B. Y. Park, Z. K. Ghouri, M. Park and H. Y. Kim, *Green Chem.,* 2015, **17**, 3791–3797.
10. N. Wang, Y. Wang, T. Guo, T. Yang, M. Chen, J. Wang, *Biosensors and Bioelectronics,* 2016, **85**, 68–75.
11. J. Shen, S. Shang, X. Chen, D. Wang, Y. Cai, *Materials Science and Engineering, 2017, 7**6,** 856–864.*
